# Deep learning on protein language model embeddings unlocks accurate prediction of protein solubility

**DOI:** 10.3389/fmicb.2026.1716930

**Published:** 2026-03-24

**Authors:** Jing Cui, Yong Jiang, Jing Wang, Han Jiang, Jiehong Fang, Jiankang Jiang, Qi Chen, Shihuan Zhong, Xinglong Wang

**Affiliations:** 1 Key Laboratory of Specialty Agri-products Quality and Hazard Controlling Technology of Zhejiang Province, College of Life Sciences, China Jiliang University, Hangzhou, Zhejiang, China; 2 School of Laboratory Medicine, Jilin Medical University, Jilin City, Jilin, China; 3 Zhejiang Gongzheng Testing Center Co., Ltd, Hangzhou, Zhejiang, China; 4 Medical Enzyme Engineering Center, CAS Key Lab of Bio-Medical Diagnostics, Suzhou Institute of Biomedical Engineering and Technology, Chinese Academy of Sciences, Suzhou, China

**Keywords:** deep learning, *Escherichia coli*, protein language models, protein solubility, synthetic biology

## Abstract

**Introduction:**

Protein misfolding is a major limitation in prokaryotic expression systems, which lack post-translational modifications and exhibit distinct intracellular environments. This severely hinders the functional expression of many heterologous proteins, especially in *Escherichia coli*. Accurate prediction of protein solubility is crucial for synthetic biology and protein engineering but remains a challenging task.

**Methods:**

Here, we present DeepSolNet, a deep learning model that leverages advanced protein language models to enhance solubility prediction. DeepSolNet adopts a multi-module architecture, integrating contextual embeddings from ESM Cambrian with bidirectional long short-term memory networks, convolutional neural networks, and attention mechanisms.

**Results:**

On the validation set, DeepSolNet achieved an accuracy of 0.75 and a Matthews correlation coefficient of 0.50 for soluble/insoluble protein classification. On an independently constructed test set containing gammabody, transglutaminase, and aldehyde dehydrogenase sequences, the model maintained high performance with an accuracy of 0.53, achieving state-of-the-art performance. Visualization analyses further showed that DeepSolNet is sensitive to key residues influencing protein solubility.

**Discussion:**

These results demonstrate that DeepSolNet serves as a powerful and generalizable tool for large-scale protein design and expression optimization. The tool is freely available at https://github.com/wangxinglong1990/DeepSolNet.

## Graphical Abstract

**Figure:**
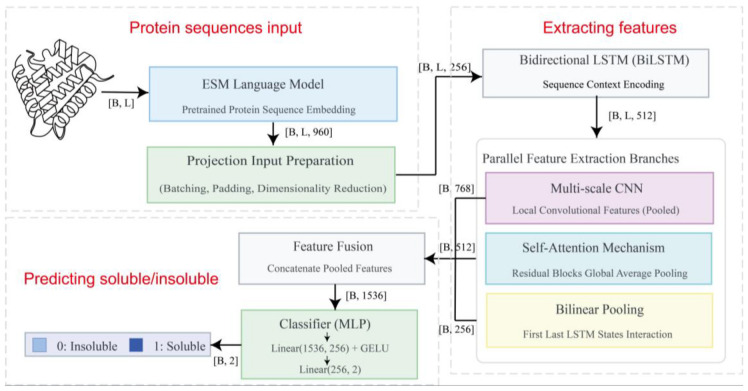
DeepSolNet is a multi-module deep learning framework for predicting protein solubility. It uses an ESM language model to generate sequence embeddings, followed by bidirectional LSTM, multi-scale CNN, and self-attention to extract features. Fused features are classified by an MLP to determine solubility (blue: insoluble, green: soluble), supporting synthetic biology and protein engineering.

## Introduction

Protein solubility refers to the ability of a protein to remain in a folded and soluble state within a given solvent, without aggregation or precipitation (Grossmann and McClements, 2023). The folding state of a protein is critical for its biological function and successful application in biotechnology and biomedicine (Kouhi et al., 2020; Sui et al., 2020). Proper folding and sufficient solubility are essential for maintaining cellular homeostasis and facilitating various life processes (Acosta-Alvear et al., 2025). In synthetic biology, there is an increasing demand for enzymes with specific catalytic functions to construct pathways. However, many heterologous proteins do not perform well in the *Escherichia coli* environment, often resulting in misfolding and loss of function (Mital et al., 2021). This is particularly true for proteins originating from eukaryotic cells that require post-translational modifications, as achieving correct folding and soluble expression in *E. coli* is challenging (İncir and Kaplan, 2024). Therefore, accurately predicting protein solubility in *E. coli* is valuable for two key reasons: first, it can help guide the selection of suitable enzymes for pathway construction (Amin et al., 2019); second, it can predict mutations that enhance the solubility of proteins, thereby increasing their production which is particularly necessary for food proteins (Yousefi and Abbasi, 2022). In both contexts, developing an accurate solubility prediction model is crucial.

Although recombinant protein expression can be achieved in various host systems, *E. coli* remains the most widely used and cost-effective host in both academic research and industrial production, owing to its fast growth, low cultivation cost, and ease of genetic manipulation (de Marco, 2025; McElwain et al., 2022). However, proteins derived from eukaryotic organisms or even from different prokaryotic species often fail to express solubly in *E. coli*. This is primarily due to differences in intracellular conditions such as redox balance, chaperone availability, and pH, which frequently result in protein misfolding and aggregation into inclusion bodies, thereby rendering the proteins insoluble and non-functional (Delivoria et al., 2025; Govers et al., 2014). Previous studies have demonstrated that fusion tags, such as soluble proteins or peptides attached to the N-terminus of the target protein, can enhance folding and solubility (Han et al., 2020; Steinmetz and Auldridge, 2017). Other approaches, including chaperone co-expression, glycosylation, and codon optimization, have also been employed to improve soluble expression (Kaur et al., 2018; Shanmugasundaram et al., 2021). However, these strategies typically rely on modifying the expression environment rather than addressing the intrinsic properties of the target protein itself. As a result, they may unintentionally alter cellular conditions in ways that negatively impact pathway efficiency or host cell growth.

To improve protein solubility by focusing on its sequence, researchers have explored computational prediction methods, including machine learning (ML) and deep learning (DL) techniques. Early methods primarily relied on statistical analysis of small-scale experimental datasets, with the advent of ML, protein solubility prediction entered a new era. Researchers began applying more sophisticated algorithms such as Support Vector Machines (SVM), Random Forests, and Gradient Boosting Machines, along with refined feature engineering to build predictive models. Traditional ML-based models, including SOLpro, PROSO II, SCM, Protein-Sol, PaRSnIP, and SoluProt, improved prediction performance by extracting hundreds or even thousands of physicochemical properties (Hebditch et al., 2017; Hon et al., 2021; Huang et al., 2012; Magnan et al., 2009; Rawi et al., 2018; Smialowski et al., 2012). However, these models heavily rely on the quality of feature selection and may not fully capture the complex, non-linear relationships hidden within sequences. In recent years, DL methods have revolutionized bioinformatics, including protein solubility prediction. Architectures like Convolutional Neural Networks (CNNs), Recurrent Neural Networks (RNNs), and their variants such as Long Short-Term Memory (LSTM) and Gated Recurrent Units (GRU) have become widely used due to their ability to automatically extract features and recognize patterns. Models like DeepSol, SKADE, EPSOL, DSResSol, and DeepSoluE are prominent examples, capable of learning deep, sequence-based features directly from amino acid sequences or their initial encodings, significantly reducing reliance on traditional feature engineering (Khurana et al., 2018; Madani et al., 2021; Raimondi et al., 2020; Wang and Zou, 2023; Wu and Yu, 2021).

This study seeks to enhance the accuracy and reliability of protein solubility prediction through two main strategies. First, we curated and thoroughly evaluated a high-quality, large-scale benchmark dataset specifically tailored for the *E. coli* expression system. This dataset integrates multiple authoritative data sources and undergoes meticulous data cleaning, de-redundancy, and standardization, providing a solid foundation for model training and evaluation, addressing the shortcomings of existing datasets. Second, based on this high-quality dataset, we developed a novel DL prediction model, DeepSolNet. The core innovation of DeepSolNet lies in its unique architecture: it leverages one of the state-of-the-art protein language models, ESM Cambrian (ESM C) (Zhang Z. et al., 2024), to extract deep, context-aware feature embeddings from input amino acid sequences. These high-quality embeddings are then processed through an integrated DL classifier that includes a Bidirectional Long Short-Term Memory network (BiLSTM) (Schuster and Paliwal, 1997), a one-dimensional Convolutional Neural Network (TextCNN) (Kim, 2014), and a self-attention mechanism (Vaswani et al., 2017). This multi-module, synergistic architecture enables DeepSolNet to capture complex sequence patterns and long-range dependencies related to protein solubility at various levels. We rigorously evaluated DeepSolNet’s performance using an independent test set, ensuring no overlap with the training data, and systematically compared it with several previously reported prediction models. Additionally, we curated a novel independent dataset from experimental data across various studies to further validate DeepSolNet’s performance. Leveraging its high accuracy, we have integrated this tool into a user-friendly web server for easy access.

## Materials and methods

### Benchmark dataset

High-quality, large-scale benchmark datasets are essential for developing robust and accurate models for protein solubility prediction. In this study, we utilized a previously curated and validated dataset, UESolDS (Zhang X. et al., 2024), specifically designed to assess the solubility of recombinant proteins expressed in *E. coli*. UESolDS was constructed by integrating data from multiple authoritative public sources, including TargetTrack (Park et al., 2024), DNASU (Seiler et al., 2014), eSOL (Delaney, 2004), and the Protein Data Bank (PDB). A rigorous six-step data cleaning pipeline was applied during dataset construction: (1) removal of 19,576 membrane proteins predicted by TMHMM; (2) exclusion of sequences containing His-tag fragments due to their uneven distribution between soluble and insoluble classes; (3) removal of 1,454 sequences containing non-standard characters; (4) filtering out 232 sequences with lengths shorter than 25 or longer than 2,500 amino acids; (5) elimination of 23,933 insoluble entries exhibiting >75% sequence identity and >70% coverage with soluble entries via BLASTp, thereby addressing data contamination; and (6) clustering of all remaining sequences using MMseqs2 at a 25% sequence identity threshold with >70% coverage to reduce sequence redundancy. After this comprehensive filtering and de-redundancy procedure, the final UESolDS dataset comprises 78,031 high-quality non-redundant protein sequences, of which 46,450 are labeled as soluble and 31,581 as insoluble.

The UESolDS dataset was divided into three non-overlapping subsets: a training set of 70,031 sequences, of which 42,450 are soluble and 27,581 are insoluble; a validation set of 4,000 sequences, of which 2,000 are soluble and 2,000 are insoluble; and an independent test set of 4,000 sequences, of which 2,000 are soluble and 2,000 are insoluble. The independent test set was further verified to share less than 25% sequence identity with the training datasets used in seven representative prior studies, including Protein_sol, SKADE, SWI, SoluProt, EPSOL, NetSolP, and DeepSoluE, enabling an unbiased evaluation of model performance (Supplementary Table 1).

### Additional external test set

To further assess the predictive capability of DeepSolNet in real-world scenarios, we curated an external test set by collecting solubility-labeled protein samples from previously published studies. This dataset includes 50 gammabody samples (Sormanni et al., 2015), eight transglutaminase samples (Wang et al., 2025), and 10 aldehyde dehydrogenase samples (Ma et al., 2025), offering a diverse and practical benchmark for evaluating model generalizability.

### Model architecture

**FIGURE 1:**
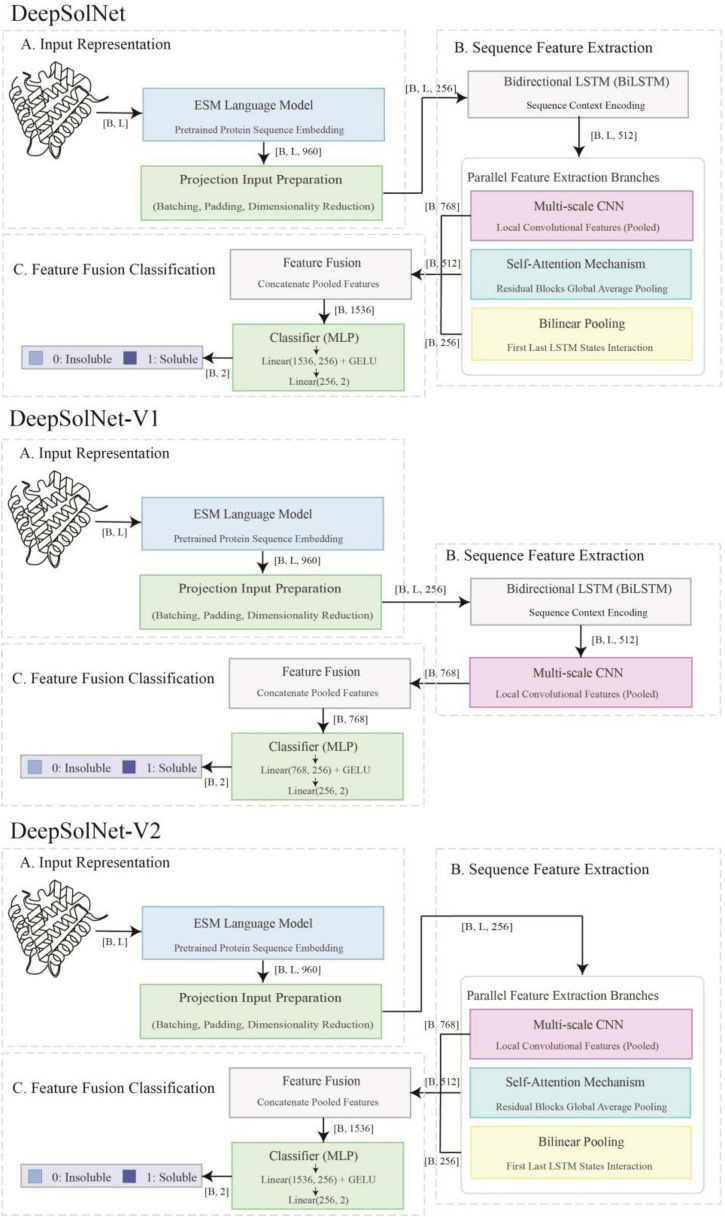
DeepSolNet architectures. Illustration of the DeepSolNet model used for predicting protein solubility. Protein sequences are first processed using the ESM language model to generate sequence embeddings, followed by feature extraction through deep learning modules. A binary classification approach is employed for the solubility prediction.

This study presents DeepSolNet, a novel DL framework designed to predict protein solubility directly from primary amino acid sequences (Figure 1). To effectively capture the intrinsic biological information encoded in protein sequences, we utilize the pre-trained protein language model ESM C, developed by Evolutionary Scale, to generate initial sequence embeddings. ESM C is a large-scale Transformer-based model trained on millions of protein sequences using an unsupervised Masked Language Modeling (MLM) objective. Through this training paradigm, ESM C learns high-dimensional contextualized representations that encode both structural and functional features of proteins. We chose ESM C as the feature extractor due to its state-of-the-art performance across various protein understanding benchmarks, making it a robust foundation for downstream prediction tasks. In DeepSolNet, each input sequence is passed through ESM C to produce an embedding matrix of shape (*B, L, D_*m*_*), where B denotes the batch size, *L* the sequence length, and *D*_*m*_ the dimensionality of the embeddings. These embeddings serve as rich input features for the subsequent modules of DeepSolNet, enabling more accurate and biologically informed solubility prediction.

The overall prediction pipeline of DeepSolNet consists of four main stages: (i) ESM C converts each input amino acid sequence into per-residue contextual embeddings; (ii) a linear projection layer maps these embeddings into a lower-dimensional, task-specific hidden space; (iii) the projected features are processed in parallel by three complementary modules — a BiLSTM network that captures long-range sequential dependencies, a TextCNN module that extracts local sequence motifs of varying lengths, and a residual attention module combined with bilinear pooling that identifies globally important positions and inter-terminal interactions; and (iv) the outputs of all modules are concatenated and passed through fully connected layers to produce the final solubility prediction. The mathematical formulations for each module are presented in the following subsections.


$$X_{e\,s\,m}\in R^{B\times L\times D_{m}}$$

The core architecture of DeepSolNet takes the protein embeddings *X*_*esm*_, generated by the ESM C model, as input and processes them through a series of specialized modules for deep feature extraction and integration, ultimately producing solubility predictions. To align feature dimensions and reduce computational complexity in subsequent layers, the raw embeddings are first passed through a linear projection layer. This layer projects the input embeddings from their original dimensionality *D*_*in*_ to a predefined hidden dimension *D*_*hid*_. Mathematically, the projection is formulated as:


$$X_{p\,r\,o\,j}=X_{e\,s\,m}\,W_{p}+b_{p}$$

Where *X*_*proj*_ ∈ *R*^*B* × *L* × *D*_*hid*_^ is a learnable weight matrix and *b*_*p*_ ∈ *R*^*D*_*hid*_^ is a bias vector. This transformation not only standardizes the embedding dimensions for downstream processing but also allows the model to learn task-specific feature representations, setting the stage for more effective deep feature extraction in the following modules.

#### BiLSTM

To capture long-range contextual dependencies in the sequence, the projected features *X*_*proj*_ are fed into an *N*_*lstm*_ layer BiLSTM network. BiLSTM processes sequence information through two parallel LSTM layers, one forward and one backward, and concatenates their hidden states at each time step t:


$$h_{t}=\left[h_{t}^{f\,w\,d};h_{t}^{b\,w\,d}\right]$$

The core computations of an LSTM unit include the input gate *i*_*t*_, forget gate *f*_*t*_, cell state update *g*_*t*_, output gate *o*_*t*_, and updates to the cell state *c*_*t*_ and hidden state *h*_*t*_, with formulas as follows:


$$i_{t}=\sigma \,\left(W_{i\,i}\,x_{t}+b_{i\,i}+W_{h\,i}\,x_{t-1}+b_{h\,i}\right)$$

$$f_{t}=\sigma \,\left(W_{i\,f}\,x_{t}+b_{i\,f}+W_{h\,f}\,h_{t-1}+b_{h\,f}\right)$$

$$g_{t}=t\,a\,n\,h\,\left(W_{i\,g}\,x_{t}+b_{i\,g}+W_{h\,g}\,h_{t-1}+b_{h\,g}\right)$$

$$O_{t}=\sigma \,\left(W_{i\,o}\,x_{t}+b_{i\,o}+W_{h\,o}\,h_{t-1}+b_{h\,o}\right)$$

$$C_{t}=f_{t}⊙C_{t-1}+i⊙g_{t}$$

$$h_{t}=o_{t}⊙\tanh\,\left(C_{t}\right)$$

The output of the BiLSTM module is *H*_*lstm*_ ∈ *R*^*B* × *L* × 2*D*_*hid*_^, which encodes context-aware features for each amino acid position.

#### TextCNN module

To extract multi-scale local sequence patterns from the BiLSTM output, we designed a TextCNN module. This module employs multiple one-dimensional convolutional layers (Conv1D) in parallel, each with different kernel sizes. The input *H*_*lstm*_ is reshaped to *B* × 2*D*_*hid*_ × *L* to suit convolutional operations. For the j-th convolutional kernel, its operation can be represented as:


$$C_{j}=G\,E\,L\,U\,\left(W_{c\,o\,n\,v_{j}}\times H_{l\,s\,t\,m}+b_{c\,o\,n\,v_{j}}\right)$$

where *W*_*conv*_*j*__ and *b*_*conv_j_*_ are the kernel weights and biases, respectively, denotes the convolution operation, and GELU (Gaussian Error Linear Unit) is used as the activation function. Subsequently, max pooling is applied along the time dimension for each convolutional output *C*_*j*_ ∈ *R*^*B* × *D*_*hid*_ × *L*^:


$$P_{j}=m\,a\,x_{t\,i\,m\,e}\,\left(C_{j}\right)$$

thus extracting a fixed-length feature vector *P*_*j*_ ∈ *R*^*B* × *D*_*hid*_^ for each convolutional kernel.

#### Residual attention module

To enable the model to dynamically focus on critical regions of the sequence and facilitate the flow of deep information, we introduced a residual module based on self-attention. This module typically contains multiple cascaded Residual Blocks, operating on the BiLSTM output *H*_*lstm*_.

#### Self-attention

The core of each residual block is a self-attention layer. The input *Y* ∈ *R*^*B* × 2*D*_*hid*_ × *L*^, where Y represents *H*_*lstm*_ or the output of the previous residual block, first undergoes Layer Normalization (Layer Norm, LN), then is linearly projected to generate Query (Q), Key (K), and Value (V) matrices:


$$Q=L\,N\,\left(Y\right)\,W_{Q},K=L\,N\,\left(Y\right)\,W_{K},V=L\,N\,\left(Y\right)\,W_{V}$$

The attention output is computed via scaled dot-product attention:


$$A\,t\,t\,e\,n\,t\,i\,o\,n\,\left(Q,K,V\right)=s\,o\,f\,t\,m\,a\,x\,\left(\frac{Q\,K^{T}}{\sqrt{d_{k}}}\right)\,V$$

where *d*_*k*_ is the dimension of the key vectors. Multi-head attention enhances expressive power by performing multiple independent attention computations in parallel and fusing their results.

#### Residual connection and feed-forward network

The output of the self-attention layer, after passing through a Dropout layer, is added to the input Y via a residual connection. Subsequently, the result undergoes another Layer Normalization and is fed into a position-wise Feed-Forward Network (FFN), which typically consists of two linear transformations and a GELU activation function:


$$Y^{{}^{\prime }}=Y+D\,r\,o\,p\,o\,u\,t\,\left(S\,e\,l\,f\,A\,t\,t\,e\,n\,t\,i\,o\,n\,\left(L\,N\,\left(Y\right)\right)\right)$$

$$H_{r\,e\,s}=Y^{{}^{\prime }}+D\,r\,o\,p\,o\,u\,t\,\left(F\,F\,N\,\left(L\,N\,\left(Y^{{}^{\prime }}\right)\right)\right)$$

where (*FFN*(*Z*)) = *GELU*(*ZW*_*f*1_ + *b*_*f*1_)*W*_*f*2_ + *b*_*f*2_

The output of the entire residual attention module, *H*_*attention*_∈*R*^*B* × 2*D*_*hid*_ × *L*^, is then subjected to average pooling, yielding *P*_*attention*_ = *mean*_*time*_(*H*_*attention*_)∈*R*^*B* × 2*D*_*hid*_^.

#### Bilinear pooling

To capture complex higher-order interactions between features at the two ends of the BiLSTM output sequence, we employed bilinear pooling. This operation acts on the hidden state of the BiLSTM output at the first time step*H*_*lstm*_0__ ∈ *R*^*B* × 2*D*_*hid*_^, and the hidden state at the last time step *H*_*lstm*_*L*−1__ ∈ *R*^*B* × 2*D*_*hid*_^:


$$P_{b\,i\,l\,i\,n}=H_{l\,s\,t\,m_{0}}^{T}\,A\,H_{l\,s\,t\,m_{L-1}}+b_{b\,i\,l\,i\,n}$$

where A is a learnable weight tensor (or a series of weight matrices corresponding to each output dimension), and *b*_*bilin*_ is a bias. The output *P*_*bilin*_ ∈ *R*^*B* × 2*D*_*hid*_^ captures specific interaction patterns of global sequence information.

#### Feature fusion and final classification

DeepSolNet effectively fuses features from different modules to form the final protein representation. Specifically, the pooled features from the TextCNN module, the pooled features from the residual attention module *p*_*att*_, and the bilinear pooling features *p*_*bilin*_ are concatenated:


$$F_{c\,o\,n\,c\,a\,t}=\left[p_{1};p_{2};\ldots ;p_{b\,i\,l\,i\,n};p_{a\,t\,t}\right]$$

The resulting fused feature vector *F*_*concat*_ ∈ *R*^*B* × *D*_*final*_^, where *D*_*final*_ is the total dimension of the concatenated features, first undergoes Layer Normalization and a Dropout layer for regularization, enhancing the model’s generalization ability:


$$F_{n\,o\,r\,m}=D\,r\,o\,p\,o\,u\,t\,\left(L\,a\,y\,e\,r\,N\,o\,r\,m\,\left(F_{c\,o\,n\,c\,a\,t}\right)\right)$$

Finally, this normalized feature vector passes through one or more Fully Connected (FC) layers for the final solubility classification. For a binary classification task, the output layer typically contains a neuron with a Sigmoid activation function (or Softmax for multi-class) or directly outputs logits for the loss function to process:


$$O_{l\,o\,g\,i\,t\,s}=D\,r\,o\,p\,o\,u\,t\,\left(G\,E\,L\,U\,\left(F_{n\,o\,r\,m}\,W_{f\,c\,1}+b_{f\,c\,1}\right)\right)\,W_{f\,c\,2}+b_{f\,c\,2}$$

where *W*_*fc*1_, *b*_*fc*1_, *W*_*fc*2_, *b*_*fc*2_ are the weights and biases of the fully connected layers.

Through the synergistic action of these multi-modules, DeepSolNet aims to learn comprehensive and discriminative feature representations from protein sequences, thereby achieving efficient and accurate prediction of protein solubility.

### Model training

The training of the DeepSolNet model follows a standard supervised learning procedure, aiming to optimize its ability to predict protein solubility. The entire process includes dataset preparation, model compilation, iterative training, and performance validation. We use protein sequence datasets in FASTA format, with each sequence associated with a binary solubility label. Data is loaded via a custom Solubility Dataset class and subjected to ESM C encoding and dynamic padding during batch processing using a collate_fn function. The model is implemented using the PyTorch framework, trained with a cross-entropy loss function and the Adam optimizer, and the learning rate is adjusted using a Reduce LROn Plateau strategy. To prevent overfitting, we implemented an early stopping mechanism and fixed random seeds to ensure experimental reproducibility.

### Evaluation metrics

To comprehensively and objectively evaluate the performance of the DeepSolNet model on the protein solubility prediction task, we employed several widely used and informative evaluation metrics in binary classification problems. These metrics are all calculated based on the four fundamental quantities of a confusion matrix: True Positives (TP), True Negatives (TN), False Positives (FP), and False Negatives (FN). In this task, “positive” typically refers to the “soluble” class.

#### Accuracy

Accuracy measures the proportion of samples correctly classified by the model out of the total number of samples, serving as the most intuitive performance metric. Its formula is:


$$\mathrm{Accuracy}=\frac{T\,P+T\,N}{T\,P+T\,N+F\,P+F\,N}$$

#### Matthews Correlation Coefficient (MCC)

Matthews Correlation Coefficient is considered a very balanced and reliable metric for evaluating the performance of binary classifiers, especially when dealing with class imbalance. It takes into account all four values of the confusion matrix, with its value ranging from −1 to +1. A +1 indicates a perfect prediction, 0 indicates a prediction no better than random guessing, and −1 indicates a prediction completely opposite to the actual outcome.

The MCC is calculated as:


$$\mathrm{MCC}=\frac{T\,P\times T\,N-F\,P\times F\,N}{\sqrt{\left(T\,P+F\,P\right)\,\left(T\,P+F\,N\right)\,\left(T\,N+F\,P\right)\,\left(T\,N+F\,N\right)}}$$

During model training, these evaluation metrics are calculated on the validation set at the end of each epoch to monitor training progress, adjust the learning rate, and decide whether to terminate training early. The finally selected model will be evaluated on an independent test set, not involved in training or validation, using the same metrics to provide an unbiased estimate of the model’s generalization ability. Additional evaluation metrics to assess the performance of the protein solubility prediction model were used, including the Area Under the Receiver Operating Characteristic curve (AUC) was used to evaluate the model’s ability to discriminate between soluble and insoluble proteins across all possible classification thresholds. A higher AUC indicates better overall model performance, as it reflects the model’s ability to correctly rank proteins based on their solubility. In addition, the Area Under the Precision-Recall curve (AUPR) was calculated to evaluate the model’s performance in scenarios with class imbalance, which is common in solubility prediction tasks.

## Results

### Architecting DeepSolNet: ablation design and component contribution analysis

To systematically investigate the contribution of each core architectural component within the DeepSolNet framework, we designed and evaluated two key ablation variants of DeepSolNet-V1 and DeepSolNet-V2 (Figure 1). By comparing these simplified versions against the complete DeepSolNet model, we aim to dissect the functional roles and relative importance of different modules in the task of protein solubility prediction.

We first introduced DeepSolNet-V1, a streamlined variant that removes two major components from the full architecture: the residual attention mechanism and the bilinear pooling layer (Figure 1). The remaining structure includes the initial projection of ESM-C embeddings, a BiLSTM module for capturing sequential dependencies, and a TextCNN module for extracting local sequence features. This design allows us to evaluate the model’s performance when it relies solely on the sequential context provided by BiLSTM and local pattern extraction by CNN, without the benefits of dynamic feature re-weighting via attention or the modeling of long-range interactions between sequence termini via bilinear pooling. The performance gap between DeepSolNet and V1 thus quantifies the collective contribution of the attention and bilinear modules to overall predictive accuracy. Next, we developed DeepSolNet-V2, which removes the BiLSTM module while retaining the residual attention and bilinear pooling components (Figure 1). In this variant, the ESM-C embeddings, after initial linear transformation, are passed directly to the TextCNN and attention layers. Since the bilinear pooling module was originally designed to operate on the first and last hidden states of BiLSTM, its use in V2 requires either modification or reinterpretation. This variant is intended to evaluate whether the rich pre-trained contextual information embedded within ESM-C representations when combined with local feature extraction and attention-based integration that can compensate for the absence of explicit long-range sequence modeling via BiLSTM.

The comparative performance of V2 against the full model provides insight into the indispensability of BiLSTM for capturing positional and sequential dependencies, which may be underrepresented or diluted in purely attention-based frameworks. Moreover, it helps clarify whether pre-trained embeddings alone are sufficient for high-accuracy solubility prediction, or if further sequence modeling remains essential. Together, these two ablation variants allow us to isolate and evaluate the functional contributions of BiLSTM, residual attention, and bilinear pooling within DeepSolNet. This layered ablation strategy provides a clearer understanding of how each component enhances the model’s predictive power and supports future optimization of similar architectures.

### Initial validation of DeepSolNet variants

**FIGURE 2:**
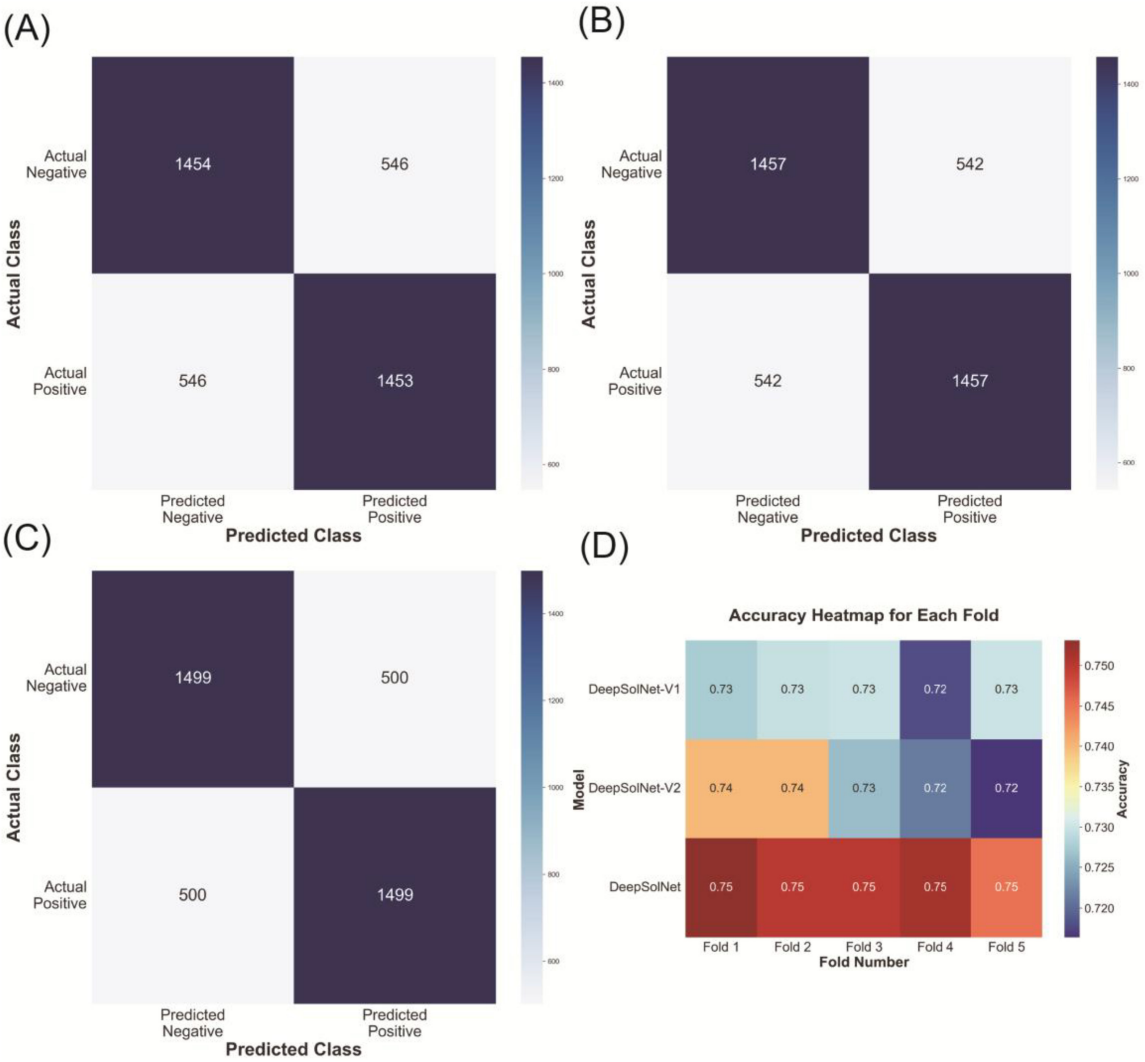
Cross-validation of DeepSolNet variants. **(A–C)** Show the best-performing fold from k-fold cross-validation for each of the three models, **(A)** V1, **(B)** V2, and **(C)** the full version. **(D)** The accuracy heatmap for DeepSolNet variants during the 5-fold validation.

To preliminarily assess the effectiveness and robustness of the DeepSolNet architecture, we conducted a 5-fold cross-validation experiment. In each iteration, the dataset was randomly partitioned into five subsets, with 4-folds used for training and the remaining one for testing. This approach ensures that the evaluation is not biased by a specific train-test split and allows for a more generalized assessment of model performance. The results consistently demonstrated that the full version of DeepSolNet outperformed both DeepSolNet-V1 and DeepSolNet-V2 in the best-performing fold (Figures 2A–C). Specifically, DeepSolNet achieved an average accuracy improvement of over 0.01 in each fold compared to the other two variants (Figure 2D). Although the numerical difference may appear modest, the consistent advantage across all test folds suggests that the full architecture exhibits superior generalization ability and robustness under varying training conditions.

### Comparison of DeepSolNet variants using independent dataset

**FIGURE 3:**
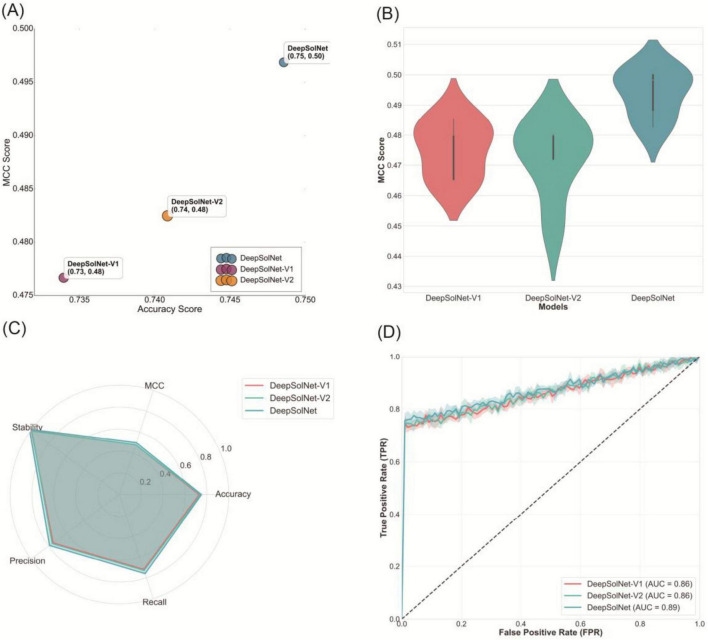
Validation of DeepSolNet variants using an independent dataset. Performance evaluation of DeepSolNet and its variants on an independent dataset comprising protein samples with less than 25% sequence identity. **(A)** Bar plots showing accuracy and Matthews Correlation Coefficient (MCC) scores for each model. **(B)** Distribution of MCC scores across all samples. **(C)** Multi-metric radar chart comparing model performance in terms of stability, precision, recall, accuracy, and MCC. **(D)** Area Under the Receiver Operating Characteristic (AUC) curve analysis demonstrating the classification performance of each variant.

To quantitatively evaluate the predictive capabilities of the model variants, we compared their performance on an independent test set using accuracy and MCC as metrics. As shown in Figure 3A, the full version of DeepSolNet outperformed both DeepSolNet-V1 and DeepSolNet-V2 in terms of both accuracy and MCC, indicating that the residual attention module, bilinear pooling layer, and BiLSTM each contribute positively to the overall performance of the model. Among the variants, DeepSolNet-V2 performed slightly better than V1, which suggests that BiLSTM-based sequence information integration plays a more critical role in this architecture than the components omitted in V1. Alternatively, it is possible that the ESM-C embeddings already provide rich sequence-level representations, allowing downstream modules to perform reasonably well even without BiLSTM. Interestingly, although DeepSolNet-V2 exhibited a broader distribution of MCC scores compared to the other models, it also produced a higher proportion of high-scoring predictions, suggesting that it may achieve greater accuracy on a subset of particularly informative or challenging samples (Figure 3B).

A multi-metric performance radar chart (Figure 3C) further demonstrated that the complete DeepSolNet architecture delivers the most balanced and superior performance across all metrics. Notably, DeepSolNet-V2 achieved higher recall and precision, reflecting enhanced predictive sensitivity and accuracy, which is particularly beneficial for capturing both soluble and insoluble proteins. In contrast, DeepSolNet-V1 exhibited higher stability, implying more consistent performance across different input conditions, potentially due to its simpler or more regularized structure. Finally, AUC curve comparisons confirmed that DeepSolNet achieved the highest AUC among the three models, further supporting the overall superiority of the full architecture (Figure 3D).

### Models comparison using independent dataset

**FIGURE 4:**
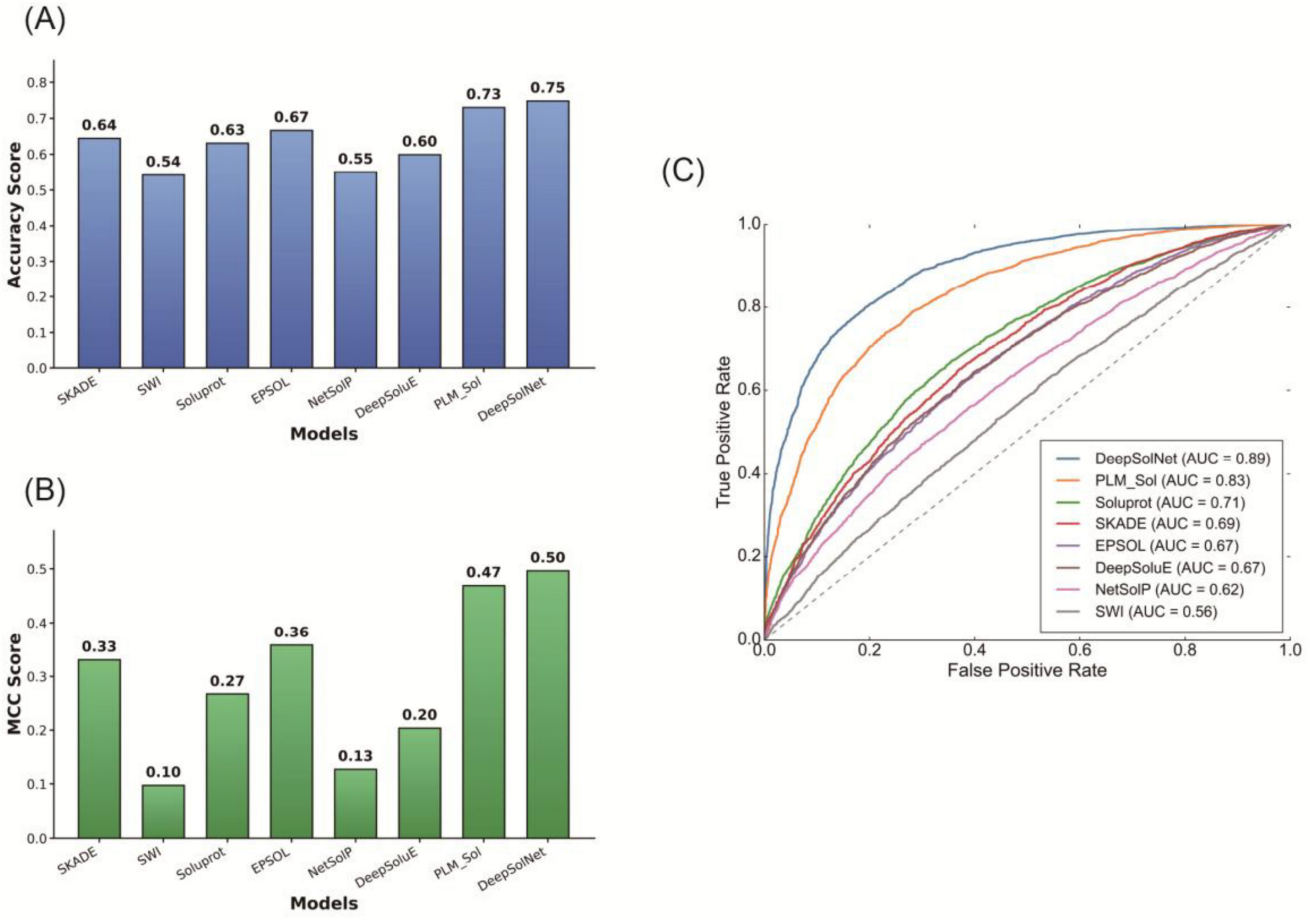
Model comparison on independent dataset. Performance comparison of various machine learning (ML) and deep learning (DL) models on the independent dataset. **(A)** Accuracy scores, **(B)** Matthews Correlation Coefficient (MCC) values, and **(C)** a comprehensive evaluation of multiple performance metrics, including accuracy, precision, recall, and MCC.

To comprehensively evaluate the performance of DeepSolNet, we conducted a systematic comparison against seven representative and previously published protein solubility prediction models: SKADE, SWI, SoluProt, EPSOL, NetSolP, DeepSoluE, and PLM_Sol, the latter also based on PLMs. All models were evaluated on the same independent test set, which shares less than 25% sequence identity with the training data of all compared models. Among them, PLM_Sol was also trained on the UESolDS dataset. Primary evaluation metrics included accuracy and MCC, supplemented by AUC, and AUPR. DeepSolNet demonstrated superior performance across all metrics. It outperformed PLM_Sol, the best-performing PLM-based baseline, with a relative Accuracy improvement of approximately 2.5% (0.75 vs. 0.73, Figure 4A). Compared to earlier ML and DL models such as SKADE, SWI, SoluProt, EPSOL, NetSolP, and DeepSoluE, DeepSolNet achieved a margin of over 0.08 in accuracy, highlighting its significant performance advantage (Figure 4A). On the more rigorous and balanced MCC metric, DeepSolNet achieved a score of 0.50, compared to 0.47 for PLM_Sol, reflecting a relative improvement of approximately 5.8% (Figure 4B), which underscores its enhanced discriminative capability for distinguishing between soluble and insoluble proteins.

Furthermore, DeepSolNet achieved the highest AUC and AUPR scores among all evaluated models (Figure 4C). Specifically, it reached an AUC of 0.89, outperforming PLM_Sol by 7.2%. In terms of precision-recall performance, DeepSolNet achieved AUPR = 0.69 and AUC = 0.56, compared to 0.62 and 0.49 by PLM_Sol, respectively. These results collectively demonstrate that DeepSolNet’s carefully engineered architecture—combining the powerful representation capabilities of the ESM C protein language model with the sequence-processing strengths of BiLSTM, TextCNN, residual attention, and bilinear pooling that sets a new benchmark in protein solubility prediction.

### Model validation on external test set

**FIGURE 5:**
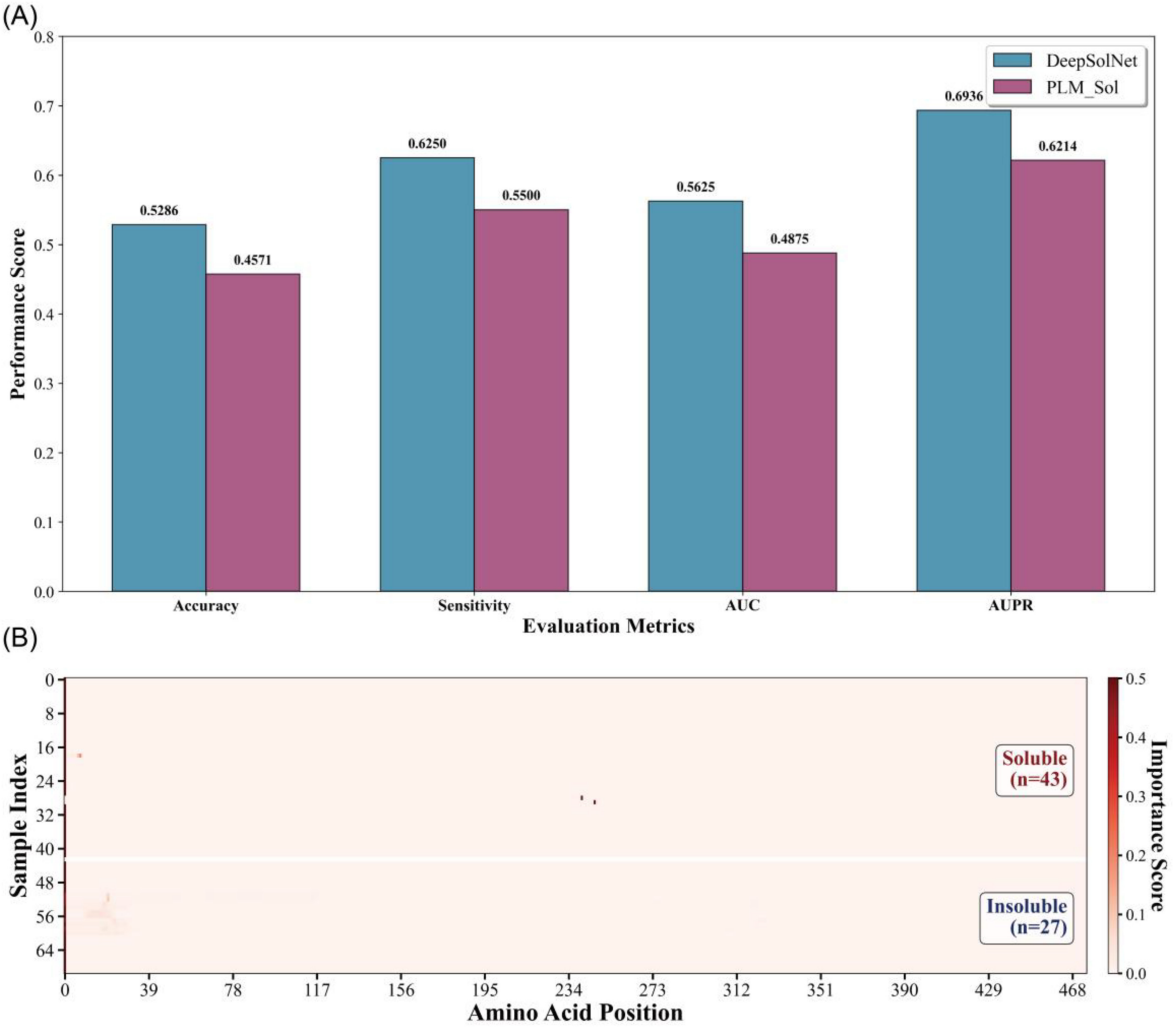
Validating performance of DeepSolNet using curated dataset. **(A)** Comparison of evaluation metrics between PLM_Sol and DeepSolNet, including accuracy, sensitivity, Area Under the Receiver Operating Characteristic curve (AUC), and Area Under the Precision-Recall curve (AUPR). **(B)** Residue-level contribution scores generated by DeepSolNet, highlighting sequence positions that influence solubility predictions. The sequence number was shown in the Supplementary materials.

To further assess the generalizability of DeepSolNet, we curated a novel dataset comprising 68 enzyme sequences, including gammabody, transglutaminase, and aldehyde dehydrogenase, with each group sharing over 70% intra-group sequence similarity. We evaluated the performance of DeepSolNet against the state-of-the-art PLM-based model PLM_Sol on this dataset. DeepSolNet achieved an accuracy of 0.53, outperforming PLM_Sol, which achieved 0.46. Additionally, DeepSolNet demonstrated superior sensitivity, AUC, and AUPR scores, underscoring its robustness and generalization capabilities (Figure 5A). Given the high sequence similarity within each enzyme group, we hypothesized that DeepSolNet may have learned to focus on specific residues that influence solubility. To investigate this, we visualized the residue-level contribution scores across the sequences to examine their influence on the overall solubility prediction. The analysis revealed that DeepSolNet could identify individual residues that significantly impact solubility (Figure 5B). However, while it could highlight influential positions, it did not consistently distinguish whether a residue had a positive or negative effect on solubility. Notably, insoluble sequences often exhibited higher contribution scores in the N-terminal region, suggesting that this region may play a critical role in solubility determination. These findings indicate that DeepSolNet is sensitive to residue-level variations and can capture localized sequence features relevant to solubility, further supporting its potential as a powerful tool for protein engineering and design.

## Discussion

In this study, we developed DeepSolNet, a DL framework for predicting protein solubility in *E. coli* by extracting informative patterns directly from primary amino acid sequences. The model integrates embeddings from the advanced protein language model ESM C with a hybrid architecture that combines BiLSTM networks, TextCNN, and attention mechanisms to capture and aggregate rich sequence features. DeepSolNet achieved an average accuracy of 0.75 in 5-fold cross-validation and reached the same accuracy on an independent test set, demonstrating robust generalization capabilities. We conducted an ablation study to evaluate the contribution of each model component and found that DeepSolNet consistently outperformed existing state-of-the-art ML and DL approaches for solubility prediction. To further validate its practical applicability, we curated additional test samples comprising gammabody, transglutaminase, and aldehyde dehydrogenase sequences from the literature. On this independent set, DeepSolNet maintained a high accuracy of 0.53. Moreover, we performed a residue-level contribution analysis, which revealed that DeepSolNet is sensitive to specific amino acids that influence solubility within a given sequence.

Recent advances in Natural Language Processing (NLP) particularly the development of Transformer-based architectures with attention mechanisms that have revolutionized the modeling of sequential data (Supriyono et al., 2024). Protein sequences often referred to as the “language of life” (Topol, 2025), share structural and hierarchical similarities with natural language, inspiring the development of PLMs such as ELMo, BERT-based variants, Transformer-XL, XLNet, and more recently, the ESM and ProtTrans series (Ofer et al., 2021). These PLMs are pre-trained on massive protein sequence databases, enabling them to capture complex long-range dependencies, evolutionary signals, and structural-functional relationships embedded within sequences, enabling the capabilities that far surpass traditional encodings like one-hot or handcrafted physicochemical descriptors (Bepler and Berger, 2021; Lupo et al., 2022). Leveraging these high-quality, context-aware embeddings has proven effective for a range of downstream prediction tasks (Masethe et al., 2024; Zeng et al., 2024). For instance, NetSolP demonstrated early success by combining ESM-1b embeddings with a multi-layer perceptron for solubility classification (Thumuluri et al., 2022). Building on these insights, our study employed ESM C as a feature extractor, which not only outperformed traditional encoding methods but also reduced feature dimensionality, enhancing computational efficiency while preserving biological relevance.

Recent studies have similarly highlighted the advantages of hybrid architectures in protein attribute prediction. For example, SoluProt utilized a ML-based framework and achieved an accuracy of 0.59 for solubility prediction (Hon et al., 2021). In contrast, PLM_Sol integrated BiLSTM and TextCNN modules to build a more expressive DL architecture, reaching 0.73 accuracy on an independent test set, outperforming traditional ML-based models (Zhang X. et al., 2024). Building upon these advancements, DeepSolNet adopts a more comprehensive and modular architecture, resulting in improved performance. The integration of BiLSTM, TextCNN, and attention mechanisms plays a pivotal role in enhancing predictive accuracy (Agbesi et al., 2024). BiLSTM captures bidirectional long-range dependencies between amino acids, which is crucial for understanding contextual sequence relationships (Gao et al., 2025). TextCNN efficiently extracts local sequence motifs associated with solubility, while the attention mechanism dynamically re-weights sequence positions to emphasize biologically relevant residues, thereby improving both model focus and interpretability (Zhang et al., 2022). Collectively, these components synergize with high-quality protein embeddings from PLMs, reinforcing the effectiveness of DeepSolNet. The model’s superior performance underscores the importance of combining multiple specialized DL modules with advanced PLM representations to achieve state-of-the-art results in protein solubility prediction.

The ablation study further clarified the role of each module within the DeepSolNet architecture. Removing either the attention and bilinear modules or the BiLSTM module resulted in consistent performance degradation across all evaluation folds and metrics. As shown in Figure 3, each simplified variant excels in a different subset of metrics, while the full architecture achieved balanced performance across all dimensions, suggesting that the modules capture complementary sequence information. In terms of overall improvement, the accuracy gain over PLM_Sol was 2.5%, while MCC and AUC showed larger improvements of 5.8% and 7.2%, respectively. The main computational cost of DeepSolNet lies in the ESM C embedding extraction, while the downstream prediction modules remain relatively lightweight.

This study introduces DeepSolNet, a DL framework designed to predict protein solubility in *E. coli* by capturing informative sequential features. The model facilitates the identification of potentially soluble enzymes, supporting rational selection for metabolic pathway construction. Moreover, DeepSolNet can assist in guiding site-directed mutagenesis by pinpointing sequence regions associated with solubility alterations. From a practical standpoint, the performance improvements achieved by DeepSolNet, particularly the 7.2% gain in AUC and 5.8% gain in MCC over PLM_Sol, are meaningful in real-world protein engineering workflows. In typical applications such as enzyme mining from metagenomic libraries or screening candidate proteins for metabolic pathway construction, researchers often rank thousands of sequences by predicted solubility and select only the top candidates for costly experimental validation. In such ranking-based scenarios, improved AUC directly translates to a higher proportion of truly soluble proteins among the selected candidates, thereby reducing the number of failed expression trials and accelerating the design-build-test cycle. Similarly, when using solubility prediction to guide site-directed mutagenesis, enhanced discriminative accuracy enables more reliable identification of beneficial mutations, reducing the experimental iterations required to achieve soluble expression. The architecture was systematically optimized from input feature extraction to the design of the predictive network, achieving an accuracy of 0.75 on an independent validation set and 0.53 on a curated experimental test set. While these results demonstrate strong predictive performance, further improvements are possible. We note that the compared models were trained on different datasets, which is a common challenge in benchmarking protein prediction tools. To mitigate this, all models were evaluated on the same independent test set with strict homology filtering, and the comparison with PLM_Sol, which shares the same training data as DeepSolNet, provides a controlled evaluation of architectural improvements. Future work should focus on expanding the training dataset through manual curation of solubility-labeled sequences from public repositories, as well as exploring more advanced network architectures to better capture subtle sequence-solubility relationships.

## Data Availability

The datasets presented in this study can be found in online repositories. The names of the repository/repositories and accession number(s) can be found in this article/Supplementary material.
