# Supplementary material for: Deep learning on protein language model embeddings unlocks accurate prediction of protein solubility
Source: Front Microbiol. 2026 Mar 24;17:1716930. doi: 10.3389/fmicb.2026.1716930 (PMC13053639; doi:10.3389/fmicb.2026.1716930)
Supplement: Supplementary file 1 [file Table_1.docx]

**Deep learning on protein language model embeddings unlocks accurate prediction of protein solubility**

Jing Cui^1,†^, Jing Wang^3^, Han Jiang^1^, Jiehong Fang^1^, Jiankang Jiang^1^, Qi Chen^1^, Shihuan Zhong^3^, Xinglong Wang^4,*^, Yong Jiang^2,†,*^

^1^ Key Laboratory of Specialty Agri-products Quality and Hazard Controlling Technology of Zhejiang Province, College of Life Sciences, China Jiliang University, 258 Xueyuan Street, Hangzhou, Zhejiang 310018, China.

^2^ School of Laboratory Medicine, Jilin Medical University, 5 Jilin Street, Jilin, Jilin 132013, China

^3^ Zhejiang Gongzheng Testing Center Co., Ltd, 128 Chengtou Street, Hangzhou, Zhejiang 311305, China.

^4^ Medical Enzyme Engineering Center, CAS Key Lab of Bio-Medical Diagnostics, Suzhou Institute of Biomedical Engineering and Technology, Chinese Academy of Sciences, Suzhou, China.

^†^. These authors contributed equally to this work, and are co-first authors.

* Corresponding author: Xinglong Wang, Yong Jiang.

Medical Enzyme Engineering Center, CAS Key Lab of Bio-Medical Diagnostics, Suzhou Institute of Biomedical Engineering and Technology, Chinese Academy of Sciences, Suzhou, China.

Phone: +86-512-69588023.

E-mail: wangxl@sibet.ac.cn, jiangyongpost@sina.com.

**Table S1: External test set**

| Sequence_ID | Reference | Protein_Name | Sequence | True_Label | Prediction | Solubility_Probability | Max_Importance_Score | Mean_Importance_Score | High_Importance_Positions |
| --- | --- | --- | --- | --- | --- | --- | --- | --- | --- |
| SIV integrase_0 | Trevino | SIV integrase | IHGQVNSDLGTWQMDCTHLEGKIVIVAVHVASGFIEAEVIPQETGRQTALFLLKLASRWPITHLHTDNGANFASQEVKMVAWWAGIEHTFGVPYNPQSQGVVEAMNHHLKNQIDRIREQANSVETIVLMAVHCMNFKRRGGIGDMTPAERLINMITTEQEIQFQ | Soluble | Soluble | 0.5684065 | 0.5012535 | 0.004198885 | [0] |
| SIV integrase_1 | Trevino | SIV integrase | IHGQVNSDLGTWQMDCTHLEGKIVIVAVHVASGFIEAEVIPQETGRQTALFLLKLASRWPITHLHTDNGANFASQEVKMVAWWAGIEHTFGVPYNPQSQGVVEAMNHHLKNQIDRIREQANSVETIVLMAVHCMNHKRRGGIGDMTPAERLINMITTEQEIQFQ | Soluble | Soluble | 0.5702151 | 0.5012526 | 0.004198443 | [0] |
| Human leptin_2 | Trevino | Human leptin | VPIQKVQDDTKTLIKTIVTRINDISHTQSVSSKQKVTGLDFIPGLHPILTLSKMDQTLAVYQQILTSMPSRNVIQISNDLENLRDLLHVLAFSKSCHLPWASGLETLDSLGGVLEASGYSTEVVALSRLQGSLQDMLWQLDLSPGC | Soluble | Soluble | 0.7510467 | 0.50124174 | 0.00457186 | [0] |
| Human leptin_3 | Trevino | Human leptin | VPIQKVQDDTKTLIKTIVTRINDISHTQSVSSKQKVTGLDFIPGLHPILTLSKMDQTLAVYQQILTSMPSRNVIQISNDLENLRDLLHVLAFSKSCHLPEASGLETLDSLGGVLEASGYSTEVVALSRLQGSLQDMLWQLDLSPGC | Soluble | Soluble | 0.80647284 | 0.50125724 | 0.004549332 | [0] |
| Human leptin_4 | Trevino | Human leptin | VPIQKVQDDTKTLIKTIVTRINDISHTQSVSSKQKVTGLDFIPGLHPILTLSKMDQTLAVYQQILTSMPSRNVIQISNDLENLRDLLHVLAFSKSCHLPQASGLETLDSLGGVLEASGYSTEVVALSRLQGSLQDMLQQLDLSPGC | Soluble | Soluble | 0.8277526 | 0.50126356 | 0.004546855 | [0] |
| Human leptin_5 | Trevino | Human leptin | VPIQKVQDDTKTLIKTIVTRINDISHTQSVSSKQKVTGLDFIPGLHPILTLSKMDQTLAVYQQILTSMPSRNVIQISNDLENLRDLLHVLAFSKSCSLPQTSGLETLDSLGEVLEASGYSTEVVALSRLQGSLQDILQQLDLSPEC | Soluble | Soluble | 0.75282604 | 0.50124615 | 0.004562835 | [0] |
| CD58_6 | Trevino | CD58 | FSQQIYGVVYGNVTFHVPSNVPLKEVLWKKQKDKVAELENSEFRAFSSFKNRVYLDTVSGSLTIYNLTSSDEDEYEMESPNITDTMKFFLYVLES | Soluble | Soluble | 0.8761499 | 0.50131214 | 0.00634676 | [0] |
| CD58_7 | Trevino | CD58 | SSQQIYGVKYGNVTFHVPSNQPLKEVLWKKQKDKVAELENSEFRAFSSFKNRVYLDTKSGSLTIYNLTSSDEDEYEMESPNITDSMKFFLYVGES | Soluble | Soluble | 0.95104134 | 0.5013025 | 0.006344166 | [0] |
| HIV type 1 integrase_11 | Trevino | HIV type 1 integrase | MHGQVDCSPGIWQLDCTHLEGKVILVAVHVASGYIEAEVIPAETGQETAYFLLKLAGRWPVKTVHTDNGSNFTSTTVKAACWWAGIKQEFGIPYNPQSQGVIESMNKELKKIIGQVRDQAEHLKTAVQMAVFIHNFKRKGGIGGYSAGERIVDIIATDIQTKE | Soluble | Soluble | 0.50300336 | 0.50126964 | 0.004208641 | [0] |
| Moloney murine leukemia virus reverse transcriptase_13 | Trevino | Moloney murine leukemia virus reverse transcriptase | TWLSDFPQAWAETGGMGLAVRQAPLIIPLKATSTPVSIKQYPMSQEARLGIKPHIQRLLDQGILVPCQSPWNTPLLPVKKPGTNDYRPVQDLREVNKRVEDIHPTVPNPYNLLSGLPPSHQWYTVLDLKDAFFCLRLHPTSQPLFAFEWRDPEMGISGQLTWTRLPQGFKNSPTLFDEALHRDLADFRIQHPDLILLQYVDDLLLAATSELDCQQGTRALLQTLGNLGYRASAKKAQICQKQVKYLGYLLKEGQRWLTEARKETVMGQPTPKTPRQLREFLGTAGFCRLWIPGFAEMAAPLYPLTKTGTLFNWGPDQQKAYQEIKQALLTAPALGLPDLTKPFELFVDEKQGYAKGVLTQKLGPWRRPVAYLSKKLDPVAAGWPPCLRMVAAIAVLTKDAGKLTMGQPLVILAPHAVEALVKQPPDRWLSNARMTHYQALLLDTDRVQFGP | Insoluble | Soluble | 0.54295695 | 0.5012751 | 0.00221232 | [0] |
| Moloney murine leukemia virus reverse transcriptase_14 | Trevino | Moloney murine leukemia virus reverse transcriptase | TWLSDFPQAWAETGGMGLAVRQAPLIIPLKATSTPVSIKQYPMSQEARLGIKPHIQRLLDQGILVPCQSPWNTPLLPVKKPGTNDYRPVQDLREVNKRVEDIHPTVPNPYNLLSGLPPSHQWYTVLDLKDAFFCLRLHPTSQPLFAFEWRDPEMGISGQLTWTRLPQGFKNSPTLFDEALHRDLADFRIQHPDLILLQYVDDLLLAATSELDCQQGTRALLQTLGNLGYRASAKKAQICQKQVKYLGYLLKEGQRWLTEARKETVMGQPTPKTPRQLREFLGTAGFCRLWIPGFAEMAAPLYPLTKTGTLFNWGPDQQKAYQEIKQALLTAPALGLPDLTKPFELFVDEKQGYAKGVLTQKLGPWRRPVAYLSKKLDPVAAGWPPCLRMVAAIAVLTKDAGKLTMGQPLVKLAPHAVEALVKQPPDRWLSNARMTHYQALLLDTDRVQFGP | Insoluble | Soluble | 0.550998 | 0.50127727 | 0.002212272 | [0] |
| Human apolipoprotein E C-terminal domain_19 | Trevino | Human apolipoprotein E C-terminal domain | KVEQAVETEPEPELRQQTEWQSGQRWELALGRFWDYLRWVQTLSEQVQEELLSSQVTQELRALMDETMKELKAYKSELEEQLTPVAEETRARLSKELQAAQARLGADMEDVCGRLVQYRGEVQAMLGQSTEELRVRLASHLRKLRKRLLRDADDLQKRLAVYQAGAREGAERGLSAIRERLGPLVEQGRVRAATVGSLAGQPLQERAQAWGERLRARMEEMGSRTRDRLDEVKEQVAEVRAKLEEQAQQIRLQAEAFQARLKSWFEPLVEDMQRQWAGLVEKVQAAVGTSAAPVPSDNH | Soluble | Soluble | 0.9296727 | 0.5012758 | 0.002772159 | [0] |
| Human apolipoprotein E C-terminal domain_20 | Trevino | Human apolipoprotein E C-terminal domain | KVEQAVETEPEPELRQQTEWQSGQRWELALGRFWDYLRWVQTLSEQVQEELLSSQVTQELRALMDETMKELKAYKSELEEQLTPVAEETRARLSKELQAAQARLGADMEDVCGRLVQYRGEVQAMLGQSTEELRVRLASHLRKLRKRLLRDADDLQKRLAVYQAGAREGAERGLSAIRERLGPLVEQGRVRAATVGSLAGQPLQERAQAWGERLRARMEEMGSRTRDRLDEVKEQVAEVRAKLEEQAQQIRLQAEAAQARLKSRFEPLAEDMQRQWAGQVEKVQAAEGTSAAPVPSDNH | Soluble | Soluble | 0.9175927 | 0.5012619 | 0.002776387 | [0] |
| Cholera toxin A1 subunit_21 | Trevino | Cholera toxin A1 subunit | NDDKLYRADSRPPDEIKQSGGLMPRGQSEYFDRGTQMNINLYDHARGTQTGFVRHDDGYVSTSISLRSAHLVGQTILSGHSTYYIYVIATAPNMFNVNDVLGAYSPHPDEQEVSALGGIPYSQIYGWYRVHFGVLDEQLHRNRGYRDRYYSNLDIAPAADGYGLAGFPPEHRAWREEPWIHHAPPGCGNAPRSS | Insoluble | Soluble | 0.8479429 | 0.5012319 | 0.003719655 | [0] |
| Cholera toxin A1 subunit_22 | Trevino | Cholera toxin A1 subunit | NDDKLYRADSRPPDEIKQSGGLMPRGQSEYFDRGTQMNINLYDHARGTQTGFVRHDDGYVSTSISLRSAHLVGQTILSGHSTYYIYVIATAPNMFNVNDVLGAYSPHPDEQEVSALGGIPYSQIYGWYRVHSGVLDEQLHRNRGYRDRYYSNLDIAPAADGYGLAGFPPEHRAWREEPWIHHAPPGCGNAPRSS | Insoluble | Soluble | 0.8349173 | 0.5012349 | 0.003717688 | [0] |
| Human translation initiation factor eIF2alpha_23 | Trevino | Human translation initiation factor eIF2alpha | MPGLSCRFYQHKFPEVEDVVMVNVRSIAEMGAYVSLLEYNNIEGMILLSELSRRRIRSINKLIRIGRNECVVVIRVDKEKGYIDLSKRRVSPEEAIKCEDKFTKSKTVYSILRHVAEVLEYTKDEQLESLFQRTAWVFDDKYKRPGYGAYDAFKHAVSDPSILDSLDLNEDEREVLINNINRRLTPQAVKIRADIEVACYGYEGIDAVKEALRAGLNCSTENMPIKINLIAPPRYVMTTTTLERTEGLSVLSQAMAVIKEKIEEKRGVFNVQMEPKVVTDTDETELARQMERLERENAEVDGDDDAEEMEAKAED | Insoluble | Soluble | 0.7880874 | 0.50135744 | 0.002670389 | [0] |
| Human translation initiation factor eIF2alpha_24 | Trevino | Human translation initiation factor eIF2alpha | MPGLSCRFYQHKFPEVEDVVMVNVRSQAEMGAYVSLLEYNNIEGMHLLSELSRRRIRSINKLIRIGRNECKVVIRVDKEKGYIDLSKRRVSPEEAIKCEDKFTKSKTVYSILRHVAEVLEYTKDEQLESLFQRTAWVFDDKYKRPGYGAYDAFKHAVSDPSILDSLDLNEDEREVLINNINRRLTPQAVKIRADIEVACYGYEGIDAVKEALRAGLNCSTENMPIKINLIAPPRYVMTTTTLERTEGLSVLSQAMAVIKEKIEEKRGVFNVQMEPKVVTDTDETELARQMERLERENAEVDGDDDAEEMEAKAED | Insoluble | Soluble | 0.7122258 | 0.50135165 | 0.002676328 | [0] |
| Human apolipoprotein D_25 | Trevino | Human apolipoprotein D | QAFHLGKCPNPPVQENFDVNKYLGRWYEIEKIPTTFENGRCIQANYSLMENGKIKVLNQELRADGTVNQIEGEATPVNLTEPAKLEVKFSWFMPSAPYWILATDYENYALVYSCTCIIQLFHVDFAWILARNPNLPPETVDSLKNILTSNNIDVKKMTVTDQVNCPKLS | Soluble | Soluble | 0.9634707 | 0.5012859 | 0.004066768 | [0] |
| Human apolipoprotein D_26 | Trevino | Human apolipoprotein D | QAFHLGKCPNPPVQENFDVNKYLGRWYEIEKIPTTFENGRCIQANYSLMENGKIKVLNQELRADGTVNQIEGEATPVNLTEPAKLEVKFSWFMPSAPYHILATDYENYALVYSCTCISQSFHVDFAWILARNPNLPPETVDSLKNILTSNNIDVKKMTVTDQVNCPKLS | Soluble | Soluble | 0.9722334 | 0.49818057 | 0.005380701 | [0, 6, 7] |
| Catalytic domain of beta4gal-T1_29 | Trevino | Catalytic domain of beta4gal-T1 | LPACPEESPLLVGPMLIEFNMPVDLELVAKQNPNVKMGGRYAPRDCVSPHKVAIIIPFRNRQEHLKYWLYYLHPVLQRQQLDYGIYVINQAGDTIFNRAKLLNVGFQEALKDYDYTCFVFSDVDLIPMNDHNAYRCFSQPRHISVAMDKFGFSLPYVQYFGGVSALSKQQFLTINGFPNNYWGWGGEDDDIFNRLVFRGMSISRPNAVVGRCRMIRHSRDKKNEPNPQRFDRIAHTKETMLSDGLNSLTYQVLDVQRYPLYTQITVDIGTPS | Insoluble | Soluble | 0.90848374 | 0.50130594 | 0.002972758 | [0] |
| Catalytic domain of beta4gal-T1_30 | Trevino | Catalytic domain of beta4gal-T1 | LPACPEESPLLVGPMLIEFNMPVDLELVEKQNPKVKTGGRYTPRDCVSPHKVAIIIPFRNRQEHLKYWLYYLHPVLQRQQLDYGIYVINQAGDTIFNRAKLLNVGFQEALKDYDYNCFVFSDVDLIPMDDHNTYRCFSQPRHISVAMDKFGFSLPYVQYFGGVSALSKQQFLTINGFPNNYWGWGGEDDDIFNRLVFRGMSISRPNAVVGRCRMIRHSRDKKNEPNPQRFDRIAHTKETMLSDGLNSLTYQVLDVQRYPLYTQITVDIGTPS | Insoluble | Soluble | 0.9425684 | 0.5013295 | 0.002963098 | [0] |
| Type S1 dihydrofolate reductase_31 | Trevino | Type S1 dihydrofolate reductase | TLSIIVAHDKQRVIGYQNQLPWHLPNDLKHIKQLTTGNTLVMARKTFNSIGKPLPNRRNVVLTNQASFHHEGVDVINSLDEIKELSGHVFIFGGQTLYEAMIDQVDDMYITVIDGKFQGDTFFPPYTFENWEVESSVEGQLDEKNTIPHTFLHLVRRKGK | Insoluble | Soluble | 0.7002408 | 0.5012659 | 0.00426422 | [0] |
| Type S1 dihydrofolate reductase_32 | Trevino | Type S1 dihydrofolate reductase | TLSIIVAHDKQRVIGYQNQLPWHLPNDLKHIKQLTTGNTLVMARKTFNSIGKPLPNRRNVVLTNQASFHHEGVDVINSLDEIKELSGHVFIFGGQTLYEAMIDQVDDMYITVIDGKFQGDTFFPPYTFEDWEVESSVEGQLDEKNTIPHTFLHLVRRKGK | Insoluble | Soluble | 0.71549124 | 0.5012049 | 0.004205119 | [0] |
| Type S1 dihydrofolate reductase_33 | Trevino | Type S1 dihydrofolate reductase | TLSIIVAHDKQRVIGYQNQLPWHLPNDLKHIKQLTTGNTLVMARKTFESIGKPLPNRRNVVLTNQASFHHEGVDVINSLDEIKELSGHVFIFGGQTLYEAMIDQVDDMYITVIDGKFQGDTFFPPYTFEDWEVESSVEGQLDEKNTIPHTFLHLVRRKGK | Insoluble | Soluble | 0.74990785 | 0.50120986 | 0.004196848 | [0] |
| Type S1 dihydrofolate reductase_34 | Trevino | Type S1 dihydrofolate reductase | TLSIIVAHDKQRVIGYQNQLPWHLPNDLKHIKQLTTGNTLVMARKTFESIGKPLPNRRNVVLTNQASFHHEGVDVINSLDEIKELSGHVFIFGGQTLYEAMIDQVDDMYITVIDGKFQGDTFFPPYTFENWEVESSVEGQLDEKNTIPHTFLHLVRRKGK | Insoluble | Soluble | 0.7258644 | 0.5012071 | 0.004200106 | [0] |
| Hemoglobin_35 | Trevino | Hemoglobin | VHLTPEEKSAVTALWGKVNVDEVGGEALGRLLVVYPWTQRFFESFGDLSTPDAVMGNPKVKAHGKKVLGAFSDGLAHLDNLKGTFATLSELHCDKLHVDPENFRLLGNVLVCVLAHHFGKEFTPPVQAAYQKVVAGVANALAHKYH | Soluble | Soluble | 0.969041 | 0.50101703 | 0.004512555 | [0] |
| Hemoglobin_36 | Trevino | Hemoglobin | VHLTPKEKSAVTALWGKVNVDEVGGEALGRLLVVYPWTQRFFESFGDLSTPDAVMGNPKVKAHGKKVLGAFSDGLAHLDNLKGTFATLSELHCDKLHVDPENFRLLGNVLVCVLAHHFGKEFTPPVQAAYQKVVAGVANALAHKYH | Soluble | Soluble | 0.9683082 | 0.5011921 | 0.004434409 | [0] |
| Hemoglobin_37 | Trevino | Hemoglobin | VHLTPEEKCAVTALWGKVNVDEVGGEALGRLLVVYPWTQRFFESFGDLSTPDAVMGNPKVKAHGKKVLGAFSDGLAHLDNLKGTFATLSELHCDKLHVDPENFRLLGNVLVCVLAHHFGKEFTPPVQAAYQKVVAGVANALAHKYH | Soluble | Soluble | 0.965719 | 0.50120157 | 0.004430707 | [0] |
| scV_tail_38 | Tan | scV_tail | MDIQMTQITSSLSASLGDRVTISCRPSQDISNFLNWYQQTPNGTVKLLIYYTSRLHSDVPSRFSGSGSGTDYSLTIDNVEQEDFATYFCQQGKTLPYTFGGGTKLEIQGGGGSGGGGSGGGGSQVQLKESGPGLVQPSQTLSITCTVSGFSLTTYGVHWLRQSPGKGLEWLGVIWSGGNTDYNAAFISRLSVNKDNSKRQVFFKLNSLQPNDTAIYYCSSNRYDWIAYWGQGTLVAVCHHHHHH | Soluble | Soluble | 0.95228684 | 0.49450722 | 0.003102132 | [240] |
| scV_tail_39 | Tan | scV_tail | MDIQMTQITSSLSASLGDRVTISCRPSQDISNFLNWYQQTPNGTVKLLIYYTSRLHSDVPSRFSGSGSGTDYSLTIDNVEQEDFATYFCQQGKTLPYTFGGGTKLEIQGGGGSGGGGSGGGGSQVQLKESGPGLVQPSQTLSITCTVSGFSLTTYGVHWLRQSPGKGLEWLGVIWSGGNTDYNAAFISRLSVNKDNSKRQVFFKLNSLQPNDTAIYYCSSNRYDWIAYWGQGTLVAVEEEEECDPHHHHHH | Soluble | Soluble | 0.95610076 | 0.47973877 | 0.003042361 | [246] |
| scV_40 | Miklos | scV | EVKLVESGGGLVDPGGSLKLECDASGFTFSSYAMSWVRQTPEKRLEWVATISTGGGYTYFPDSVKGRFTISRDNAKNALYLQMKSLRSEDTADYYCARQGDFGDWYFDVWGAGTTVTVSGSGSGSGSGDVLMTQTPLSLPVELGDQASIECRSSQSLVHSNGNTYLHWYLQKPGQSPKLLIYKVSNRFSGVPDRFSGSGSGTDFTLKIDRVEAEDLGVYFCSQSTHVPWTFGGGTKLEIKRA | Insoluble | Soluble | 0.7901818 | 0.5012411 | 0.003122405 | [0] |
| scV_41 | Miklos | scV | EVKLDESGGGEVDPGGSLKLECDASGFTFSSYAMSWVRQTPEKRLEWVATISTGGGYTYFPDSVKGRFTIDRDNDKNALYLQMKSLRSEDTADYYCARQGDFGDWYFDVWGAGTTVTVSGSGSGSGSGDVLMTQTPDSLPVELGDQASIECRSSQSLVHSNGNTYLHWYLQKPGQSPKLLIYKVSNRFSGVPDRFSGSGSGTDFELKIDEVEAEDLGVYFCSQSTHVPWTFGGGTKLEIKRA | Insoluble | Soluble | 0.85622716 | 0.50122887 | 0.003117816 | [0] |
| scV_42 | Miklos | scV | EVKLDESGGGEVDPGGSLKLECDASGFTFSSYAMSWVRQTPEDRLEWVATISTGGGYTYFPDSVKGRFEIDRDNDKNALYLQMKSLRDEDTADYYCARQGDFGDWYFDVWGAGTTVTVSGSGSGSGSGDVLMTQTPDELPVELGDQASIECRSSQSLVHSNGNTYLHWYLQKPGQSPKLLIYKVSNRFSGVPDRFSGSGSGTDFELKIDEVEAEDLGVYFCSQSTHVPWTFGGGTKLEIKRA | Insoluble | Soluble | 0.895274 | 0.5012264 | 0.003117041 | [0] |
| scV_43 | Miklos | scV | EVKLDEDGGGEVDPGGSLKLECDADGFTFSSYAMSWVRQTPEDRLEWVATISTGGGYTYFPDSVKGRFEIDRDNDKNALYLQMKSLRSEDTADYYCARQGDFGDWYFDVWGAGTTVTVSGSGSGSGSGDVLMEQTPDELPVEEGDEASIECRSSQSLVHSNGNTYLHWYLQKPGQSPKLLIYKVSNRFSGVPDRFDGSGDGTDFTLKIDEVEEEDLGVYFCSQSTHVPWTFGGGTKLEIKRA | Insoluble | Soluble | 0.9222265 | 0.50123346 | 0.003118477 | [0] |
| scV_44 | Miklos | scV | EVKLVESGGGLVKPGGSLKLKCKASGFTFSSYAMSWVRQRPEKRLEWVATISTGGGYTYFPDSVKGRFKISRDNAKNALYLQMKSLRSEDTARYYCARQGDFGDWYFDVWGAGTTVTVSGSGSGSGSGDVLMTQTPLKLPVSLGDQASIKCRSSQSLVHSNGNTYLHWYLQKPGQSPKLLIYKVSNRFSGVPDRFSGSGSGTDFTLKIKRVEAEDLGVYFCSQSTHVPWTFGGGTKLEIKRA | Soluble | Soluble | 0.7191877 | 0.5012153 | 0.003143419 | [0] |
| scV_45 | Miklos | scV | EVKLVESGGGKVKPGGSLKLKCKASGFTFSSYAMSWVRQRPEKRLEWVATISTGGGYTYFPDSVKGRFKISRDNAKNALYLQMKSLRSEDTARYYCARQGDFGDWYFDVWGAGTTVTVSGSGSGSGSGDVLMTQTPKKLPVSLGDQASIRCRSSQSLVHSNGNTYLHWYLQKPGQSPKLLIYKVSNRFSGVPDRFSGSGSGTDFRLKIKRVEAEDLGVYFCSQSTHVPWTFGGGTKLEIKRA | Soluble | Soluble | 0.73751193 | 0.5012147 | 0.003146913 | [0] |
| scV_46 | Miklos | scV | KVKLVESGGGKVKPGGSLKLKCKASGFTFSSYAMSWVRQRPEKRLEWVATISTGGGYTYFPDSVKGRFKISRDNAKNALYLQMKSLRKEDTARYYCARQGDFGDWYFDVWGAGTTVTVSGSGSGSGSGDVLMTQTPKKLPVRLGDQASIRCRSSQSLVHSNGNTYLHWYLQKPGQSPKLLIYKVSNRFSGVPDRFSGSGSGTDFRLKIKRVEAEDLGVYFCSQSTHVPWTFGGGTKLEIKRA | Soluble | Soluble | 0.7857831 | 0.5012293 | 0.003149028 | [0] |
| scV_47 | Miklos | scV | KVKLVESGGGRVKPGGSLKLKCKASGFTFSSYAMSWVRQRPEKRLEWVATISTGGGYTYFPDSVKGRFKISRDNKKNKLKLKMKSLRKEDTARYYCARQGDFGDWYFDVWGAGTTVTVSGSGSGSGSGDVLMTQRPKKLPVRLGDKASIRCRSSQSLVHSNGNTYLHWYLQKPGQSPKLLIYKVSNRFSGVPDRFSGSGSGTDFRLKIKRVEKEDLGVYFCSQSTHVPWTFGGGTKLEIKRA | Soluble | Soluble | 0.8463379 | 0.50123155 | 0.003159975 | [0] |
| scV_48 | Miklos | scV | RVKLVESGGGRVKPGGRLKLSCRASGFTFSSYAMSWVRQRPEKRLEWVATISTGGGYTYFPDSVKGRFRIRRDNKKRRLYLQMKSLRREDKARYYCARQGDFGDWYFDVWGAGTTVTVSGSGSGSGSGDVLMRQRPRSLPVRKGDKASIRCRSSQSLVHSNGNTYLHWYLQKPGQSPKLLIYKVSNRFSGVPDRFSGSGRGRDFRLKIKRVEKEDLGVYFCSQSTHVPWTFGGGTKLEIKRA | Insoluble | Soluble | 0.86846036 | 0.50123084 | 0.003164388 | [0] |
| scV_49 | Miklos | scV | EVKLVESGGGLVKPGGSLKLSCAASGFTFSSYAMSWVRQTPEKRLEWVATISTGGGYTYFPDSVKGRFTISRDNAKNALYLQMKSLRSEDTAMYYCARQGDFGDWYFDVWGAGTTVTVSGSGSGSGSGDVLMTQTPLSLPVSLGDQASISCRSSQSLVHSNGNTYLHWYLQKPGQSPKLLIYKVSNRFSGVPDRFSGSGSGTDFTLKISRVEAEDLGVYFCSQSTHVPWTFGGGTKLEIKRA | Insoluble | Soluble | 0.7255354 | 0.5012362 | 0.003138003 | [0] |
| Streptomyces mobaraenesis_58 | Streptomyces mobaraenesis | Streptomyces mobaraenesis | DNGAGEETKSYAETYRLTADDVANINALNESAPAASSAGPSFRAPDPDDRVTPPAEPLDRMPDPYRPVNGRAETVVNNYIRKWQQVYSHRDGRKQQMTEEQREWLSYGCVGVTWVNSGQYPTNRLAFASFDEDRFKNELKNGRPRSGETRAEFEGRVAKESFDEEKGFQRAREVASVMNRALENAHDESAYLDNLKKELANGNDALRNEDARSPFYSALRNTPSFKERNGGNHDPSRMKAVIYAKHFWSGQDRSSSADKRKYGDPDAFRPAPGTGLVDMSRDRNIPRSPTSPGEGFVNFDYGWFGAQTEADADKTVWTHGNHYHAPNGSLGAMHVYESLFRNWSEGYSDFDRGAYVITFIPKSWNTAPDKVKQGWP | Soluble | Soluble | 0.67591155 | 0.50117314 | 0.002385709 | [0] |
| EHW8109330.1_60 | EHW8109330.1 | EHW8109330.1 | MNQQDIEQVVKAVLLKMKDSSQPASTVHEMGVFASLDDAVAAAKRAQQGLKSVAMRQLAIHAIREAGEKHARELAELAVSETGMGRVDDKFAKNVAQARGTPGVECLSPQVLTGDNGLTLIENAPWGVVASVTPSTNPAATVINNAISLIAAGNSVVFAPHPAAKKVSQRAITLLNQAVVAAGGPENLLVTVANPDIETAQRLFKYPGIGLLVVDAARKHTNKRLIAAGAGNPPVVVDETADLPRAAQSIVKGASFDNNIICADEKVLIVVDSVADELMRLMEGQHAVKLTAAQAEQLQPVLLKNIDERGKGTVSRDWVGRDAGKIAAAIGLNVPDQTRLLFVETPANHPFAVTEMMMPVLPVVRVANVEEAIALAVQLEGGCHHTAAMHSRNIDNMNQMANAIDTSIFVKNGPCIAGLGLGGEGWTTMTITTPTGEGVTSARTFVRLRRCVLVDAFRIVLE | Soluble | Soluble | 0.54003054 | 0.501204 | 0.00213063 | [0] |
| EFB1091884.1_61 | EFB1091884.1 | EFB1091884.1 | MNQQDIEQVVKAVLLKMQSSDTPSAAVHEMGVFASLDDAVAAAKVAQQGLKSVAMRQLAIAAIREAGEKHARDLAELAVSETGMGRVEDKFAKNVAQARGTPGVECLSPQVLTGDNGLTLIENAPWGVVASVTPSTNPAATVINNAISLIAAGNSVIFAPHPAAKKVSQRAITLLNQAIVAAGGPENLLVTVANPDIETAQRLFKFPGIGLQVVTGGEAVVEAARKHTNKRLIAAGAGNPPVVVDETADLARAAQSIVKGASFDNNIICADEKVLIVVDSVADELMRLMEGQHAVKLTAEQAQQLQPVLLKNIDERGKGTVSRDWVGRDAGKIAAAIGLKVPQETRLLFVETTAEHPFAVTELMMPVLPVVRVANVADAIALAVKLEGGCHHTAAMHSRNIENMNQMANAIDTSIFVKNGPCIAGLGLGGEGWTTMTITTPTGEGVTSARTFVRLRRCVLVDAFRIVLE | Soluble | Soluble | 0.5231586 | 0.50120044 | 0.002114258 | [0] |
| HhaI methyltransferase_8 | Trevino | HhaI methyltransferase | MIEIKDKQLTGLRFIDLFAGLGGFRLALESCGAECVYSNEWDKYAQEVYEMNFGEKPEGDITQVNEKTIPDHDILCAGFPCQAFSISGKQKGFEDSRGTLFFDIARIVREKKPKVVFMENVKNFASHDNGNTLEVVKNTMNELDYSFHAKVLNALDYGIPQKRERIYMICFRNDLNIQNFQFPKPFELNTFVKDLLLPDSEVEHLVIDRKDLVMTNQEIEQTTPKTVRLGIVGKGGQGERIYSTRGIAITLSAYGGGIFAKTGGYLVNGKTRKLHPRECARVMGYPDSYKVHPSTSQAYKQFGNSVVINVLQYIAYNIGSSLNFKPY | Insoluble | Insoluble | 0.3632096 | 0.5012611 | 0.002644545 | [0] |
| HhaI methyltransferase_9 | Trevino | HhaI methyltransferase | MIEIKDKQLTGLRFIDLFAGLGGFRLALESCGAECVYSNEWDKYAQEVYEMNFGEKPEGDITQVNEKTIPDHDILCAGFPCQAFSISGKQKGFEDSRGTLFFDIARIVREKKPKVVFMENVKNFASHDNGNTLEVVKNTMNELDYSFHAKVLNALDYGIPQKRERIYMICFRNDLNIQNFQFPKPFELNTFVKDLLLPDSEVEHLVIDRKDLSMTNQEIEQTTPKTVRLGIVGKGGQGERIYSTRGIAITLSAYGGGIFAKTGGYLVNGKTRKLHPRECARVMGYPDSYKVHPSTSQAYKQFGNSVVINVLQYIAYNIGSSLNFKPY | Insoluble | Insoluble | 0.36127758 | 0.5012603 | 0.002644868 | [0] |
| HhaI methyltransferase_10 | Trevino | HhaI methyltransferase | MIEIKDKQLTGLRFIDLFAGLGGFRLALESCGAECVYSNEWDKYAQEVYEKNFGEKPEGDITQVNEKTIPDHDILCAGFPCQAFSISGKQKGFEDSRGTLFFDIARIVREKKPKVVFMENVKNFASHDNGNTLEVVKNTMNELDYSFHAKVLNALDYGIPQKRERIYMICFRNDLNIQNFQFPKPFELNTFVKDLLLPDSEVEHLVIDRKDLVMTNQEIEQTTPKTVRLGIVGKGGQGERIYSTRGIAITLSAYGGGIFAKTGGYLVNGKTRKLHPRECARVMGYPDSYKVHPSTSQAYKQFGNSVVINVLQYIAYNIGSSLNFKPY | Insoluble | Insoluble | 0.3616733 | 0.50126255 | 0.002644761 | [0] |
| HIV type 1 integrase_12 | Trevino | HIV type 1 integrase | MHGQVDCSPGIWQLDCTHLEGKVILVAVHVASGYIEAEVIPAETGQETAYFLLKLAGRWPVKTVHTDNGSNFTSTTVKAACWWAGIKQEFGIPYNPQSQGVIESMNKELKKIIGQVRDQAEHLKTAVQMAVFIHNKKRKGGIGGYSAGERIVDIIATDIQTKE | Soluble | Insoluble | 0.49997643 | 0.50126946 | 0.004210219 | [0] |
| Xylose isomerase_15 | Trevino | Xylose isomerase | MAYFNDIAPIKYEGTKTKNMFAFRHYNPEEVVAGKTMEEQLHFALAFWHTITMDGSDPFGGATMERPWDLEGGSELDRAHRRVDAFFEIAEKLGVKYYCFHDIDIAPTGNSLKEFYANLDEITDHLLEKQKATGIKLLWNTANMFSNPRYMNGVSTSNRAEVFAYGAAQVKKGLELSKKLGGENYVFWGGREGYESLLNTDRGLEMDHMAKFFHLAIYYAKSINHLPIFLIEPKPKEPMTHQYDFDAATALAFLQKYDLDKYFKLNLETNHAWLVGHTFEHELNTARTFNALGSIDANQGNYLLGWDTDEFPTLVIDITLAMHQILLNGGLGKGGINFDAKVRRTSFKAEDLILAHIAGMDTYARALKGAAAIIEDKFLSDIVDERYSSYKNTEVGQSIENGTATFKSLAAFALEHGDDIELDSNHLEYIKSVLNDYLV | Insoluble | Insoluble | 0.44571993 | 0.5012737 | 0.00224265 | [0] |
| Xylose isomerase_16 | Trevino | Xylose isomerase | MAYFNDIAPIKYEGTKTKNMFAFRHYNPEEVVAGKTMEEQLHFALAFWHTITMDGSDPFGGATMERPWDLEGGSELDRAHRRVDAFFEIAEKLGVKYYCFHDIDIAPTGNSLKEFYANLDEITDHLLEKQKATGIKLLWNTANMFSNPRYMNGVSTSNRAEVFAYGAAQVKKGLELSKKLGGENYVFWGGREGYESLLNTDMGLEMDHMAKFFHLAIDYAKSINHLPIFLIEPKPKEPMTHQYDFDAATALAFLQKYDLDKYFKLNLETNHAWLAGHTFEHELNTARTFNALGSIDANQGNYLLGWDTDEFPTLVIDITLAMHQILLNGGLGKGGINFDAKVRRTSFKAEDLILAHIAGMDTYARALKGAAAIIEDKFLSDIVDERYSSYKNTEVGQSIENGTATFKSLAAFALEHGDDIELDSNHLEYIKSVLNDYLV | Insoluble | Insoluble | 0.45315439 | 0.5012819 | 0.002242464 | [0] |
| Xylose isomerase_17 | Trevino | Xylose isomerase | MAYFNDIAPIKYEGTKTKNMFAFRHYNPEEVVAGKTMEEQLHFALAFWHTITMDGSDPFGGATMERPWDLEGGSELDRAHRRVDAFFEIAEKLGVKYYCFHDIDIAPTGNSLKEFYANLDEITDHLLEKQKATGIKLLWNTANMFSNPRYMNGVSTSNRAEVFAYGAAQVKKGLELSKKLGGENYVFWGGREGYESLLNTDRGLEMDHMAKFFHLAIYYAKSINHLPIFLIEPKPKEPMTHQYDFDAATALAFLQKYDLDKYFKLNLETNHAWLVGHTFEHELNTARTFNALGSIDANQGNYLLGWDTDEFPTLVIDITLAMHQILLNGGLGKGGINFDAKVRRTSFKAEDLILAHIAGMDTYARALKGAAAIIEDKFLSDIVDERYSSYKNTEVGQSIENGTATFESLAAFALEHGDDIELDSNHLEYIKSVLNDYLV | Insoluble | Insoluble | 0.4530268 | 0.50127727 | 0.002242569 | [0] |
| Xylose isomerase_18 | Trevino | Xylose isomerase | MAYFNDIAPIKYEGTKTKNMFAFRHYNPEEVVAGKTMEEQLHFALAFWHTITMDGSDPFGGATMERPWDLEGGSELDRAHRRVDAFFEIAEKLGVKYYCFHDIDIAPTGNSLKEFYANLDEITDHLLEKQKATGIKLLWNTANMFSNPRYMNGVSTSNRAEVFAYGAAQVKKGLELSKKLGGENYVFWGGREGYESLLNTDRGLEMDHMAKFFHLAIYYAKSINHLPIFLIEPKPKEPMTHQYDFDAATALAFLQKYDLDKYFKLNLETNHAWLVGHTFEHELNTARTFNALGSIDANQGNYLLGWDTDEFPTLVIDITLAMHQILLNGGLGKGGINFDAKVRRTSFKAEDLILAHIAGMDTYARALKGAAAIIEDKFLSDIVDERYTSYKNTEVGQSIENGTATFKSLAAFALEHGDDIELDSNHLEYIKSVLNDYLV | Insoluble | Insoluble | 0.44649345 | 0.5012742 | 0.002242624 | [0] |
| Potassium channel KcsA_27 | Trevino | Potassium channel KcsA | MPPMLSGLLARLVKLLLGRHGSALHWRAAGAATVLLVIVLLAGSYLAVLAERGAPGAQLITYPRALWWSVETATTVGYGDLYPVTLWGRLVAVVVMVAGITSFGLVTAALATWFVLREQERRGHFVRHSEKAAEEAYTRTTRALHERFDRLERMLDDNRR | Insoluble | Insoluble | 0.17862625 | 0.38780838 | 0.006010306 | [0, 20] |
| Potassium channel KcsA_28 | Trevino | Potassium channel KcsA | MPPMLSGLLARLVKLLLGRHGSADHERAAGAATVLLVIVLLAGSYLAVLAERGAPGAQLITYPRALWWSVETATTVGYGDRYPVTLWGRLVAEVVMVAGITSFGLVTAALATWFVRREQERRGHFVRHSEKAAEEAYTRTTRALHERFDRLERMLDDNRR | Insoluble | Insoluble | 0.19708961 | 0.39423773 | 0.00506873 | [0, 20] |
| Streptomyces sp. CG 926_50 | Streptomyces sp. CG 926 | Streptomyces sp. CG 926 | MYKRSGFLALTTVGVVLCTAGLAPSVSQAAGSADGGSGSYAETHGLTVDDIGHINALNEKALTAGRPDDPTGGLPPSATESHGASTSADDRVTPPAEPLGSMPDAYRARGDRAATGISNYIRKWQQTYSHRDGRPQQMTQAQREQLSYGCVGVTWVNSGPYPTNKLAFASFDEDRYKDALENTGPRPGETRAEFEGRIAKQSFDETKGFDRARDVASVMNKALENAHDEGTYLSNLKAELTKKDDALAGEDSRSNFYSALRNTPSFKDRNGGNLDPSTMKAVIYSKHFWSGQDPRASFDKRKYGDPEAFRPDRNTGLVDMSKDRNTSRSPAKPGESYVNFDYGWFGDQKETAPDNTIWTHANHYHSPGGAMGPMQVYESKFRSWSSGYTDFDRGTYVVTFIPKSWNTAPAKVKQGWP | Soluble | Insoluble | 0.18821965 | 0.4339974 | 0.00239154 | [0, 18, 19] |
| Streptomyces sp. H021_51 | Streptomyces sp. H021 | Streptomyces sp. H021 | MYKRSGFLALTTAGIVLCTAGLTPSVSQAAGGTGDGKGSYADSNGLTADDIRLINALNEEALDLGRPGKTPGGDLPSATESLGAPTAADDRVTPPAEPLDRLPDAYRAHGDRATTGISNYIRKWQQVYSHRDGRAQQMTEQQREQLSYGCVGVTWVNSGPYPTNKLAFASFDEEKHRNALENTSPRPGETRAEFEGRIAKQSFDEAKGFNRARDVASVMNKALDNAHDETTYLSNLKAELARNDDALAAEDSRSNFYSALRNTPSFKDRNGGNHDPSRMKAVIYSKHFWSGQDPRSSSDKRKYGDPDAFRPNRNTGLVDMSKDRNTSRSPARPGESYVNFDYGWFGDQKETNADNTIWTHANHYHSPEGGMGPMQVYESKFRSWSSGYSDFDRGTYVITFIPKNWNTAPAKVKQGWP | Insoluble | Insoluble | 0.20826529 | 0.47459424 | 0.00239417 | [0, 18, 19] |
| Streptomyces sp. HA201_52 | Streptomyces sp. HA201 | Streptomyces sp. HA201 | MSQRRKFLAFATVGAVMCTAGFMPTVSQAAGNSEGGEKEGSYAETHDLTVADVKSINALNARGLAVGQPGKPRAEFPPGASSLFRAADDRVTPPAEPLNRMPDAYQPYGGRATTVVGNYIRKWQQVYSQRDGKAQQMTAEQREKLSYGCVGVTWANSGPYPTNSLAFASFDENKYKNDLKNTRPRSGETQAEFEGRIAKDNFDEAKGFKRARDVASTMNKALENAHDEGTYLNNLKTELSNSKDALLYEDSRSNFYSALRNTPSFKERDGGNYDPSKMKAVVYSKHFWSGQDSRSSSEKRKFGDPDAFRPNQGTGLVDMSQDRNVPRSPANPGEGWVNFDYGWFGAQTEADADKTIWTHANHYHAPNSDLGAMQVYESKFRNWSAGYADFDRGAYMITFIPKSWNTAPAKVMQGWS | Insoluble | Insoluble | 0.28685772 | 0.48521024 | 0.002397044 | [0, 11, 12, 13, 14, 15, 16, 17, 18, 19, 20] |
| Streptomyces sp. NRRL_53 | Streptomyces sp. NRRL | Streptomyces sp. NRRL | MQKRRGFMAFTAASVAMCTIGFLPTVSHAAEIGPRGGNGSYAARNGLTANDVRSINALNEAALNLGQPGRPSPSTSSPFGALGDDRVTPPAEPLNLLPSPYRAFNGRASTGVSNYIRKWQQVYSQRDGKMQQMTEQQREELSYGCVGVTWVNSGSYPTNNLAFASFDETKYKNSLANSTRRPGETQAEFEGRIAKSSFDEKKGFERARNVTATINKALENAHSETDYLTNLKADLTSKGDALANQDSRSNFYSALRNTASFKDKNGGNFDPSKMKAVIYSKHFWSGQDPWSSPEKSKFGDPNGFRPDRATGLVDMSQDWNMSRSAVKEGDAYVNFDYGWFGDQTETDANKTIWTHANHYNSPGGDMGPMDVYESTFPNWASGYEDFDRGAYMVVFIPKSWNTAPAKVKEGWQ | Soluble | Insoluble | 0.26021543 | 0.48436505 | 0.002423631 | [0, 10, 11, 12, 13, 14, 15, 16, 17, 18, 19, 20, 21] |
| Actinobacteria bacterium OV450_54 | Actinobacteria bacterium OV450 | Actinobacteria bacterium OV450 | MYTRSKFLTFTAVAVVMCTAGLVPSVGHAAEVPTGVPAGETVRSYAENHGLTAADVMRINTLNEKALSLGQPGKPTGSVPPNASESSRAAADGGMETPPAEPLERMPDPYRVYGGRASTSVGNYIRKWQQVYSQRDGQRQQMTEAQRQALAYGCVGVTWVNSGNYPTNKLGFAFFDESKYKDAVKNTSPRPGETQSEFEGRVAKQSFDEEKGFKRARDVASVMNKALEGSGDENAYLTKLKAELGKTNDALADEDGRSNFYAALRNTPSFRDKNGGNSDPSKMKAVIYSKHFWSAQDPRSSADNRKFGDPEGFRPNQSTGLVDMSKDRNTSRSATKPGESFVNFDYGWFGDQKEPAADKTIWTHANHFHNPNGDMGPMNVYESRFQNWSSGYADFDRGAYMVTFIPKSWNTAPDNVTQGWR | Insoluble | Insoluble | 0.18904734 | 0.43426356 | 0.002365791 | [0, 23] |
| Streptomyces roseoverticillatus_55 | Streptomyces roseoverticillatus | Streptomyces roseoverticillatus | MYKRQRILTFATAGAVICTAGFIPSVSQAAGSGDREEKGSYAETHGLTADDVKNINELNESALAPGQPGKPPGELPPSAGPSSRAPGSTDDRETPPAEPLERMPDAYRAYGGRATTVINNYIRKWQQVYSHRDGKKQQMTEEQREKLSYGCVGVTWANSGPYPMNKLAFASFDENKYKNDLKNTSPRPDETRAEFEGRIAKDSFDEEKGFKRARDVASIMNKALESAHDEGTYIDNLKTGLTNNNDALLHEDSRSNFYSALRNTPSFKERDGGNYDPSKMKAVIYSKHFWSGQDQRSPSDRRKYGDPEAFRPDQGTGLVDMSKDRSIPRSPANPGESWVNFDYGWFGAQTEADADKTVWTHGDHYHAPNSGLGPMHVYESKFRNWSAGYADFDRGTYMITFIPKSWNTAPAKVQQGWP | Soluble | Insoluble | 0.22450294 | 0.48049438 | 0.00239004 | [0, 24] |
| Streptomyces sp. TN58_56 | Streptomyces sp. TN58 | Streptomyces sp. TN58 | MYKRSGFLALATAGMVLCTAGLAPSVSQAAGAAPVRGDGKGSYAETNGLTAEDIRLINTLNEKALDLGRPDKTAGEGLPNATASLRPSAAAGGRVTPPAEPLDRMPDPYRAHGDRATTGISNYIRKWQQAYSHRDGQARQMTGQQREQLSYGCVGVTWVNSGPYPTNRLAFASFDEDKHKNALENTSPRPGETRAEFEGRIAKQSFDEAKGFNRARDVAAVMNKALENSHDENAYLNNLKAELTKKNDALAGEDSRSNFYAALRNTPSFWDSSGGDRDPSKMKAVIYSKHFWSGQDPWSPADKRKYGDPDAFRPDRATGLVDMSKDRNTSRSPAKPGEGYVNFDYGWFGDQKEANPDHTIWTHANHYHSPEGGMGPMQVYESKFRSWSAGYTDFDRGTYVITFIPKSWNTAPAKVKQGWP | Soluble | Insoluble | 0.19155084 | 0.4297258 | 0.002376101 | [0, 18, 19] |
| Streptomyces wuyuanensis_57 | Streptomyces wuyuanensis | Streptomyces wuyuanensis | MHTRSRFLAFTTVGVVMCTAGFIPSVSQAAHRVEGEERESFAKEHGLTADDVRHINALNEKALSLGLPGKPSGASLPSAIESLGASAAASGRVTPPAEPLDSMPDAYRTYGGRATTGVSNYIRKWQQVYSHRDGQARQMTEEQREQLSYGCVGVTWVNSGPYPTNKLAFASFDENKYKNALEHTTPRPGETRAELEGRIAKESFDEGKGFKRARDVASIMNKALENAHDEGTYLNNLKVELTRSNDALRNEDARSNFYSALRNTPSFKNKDGGNHDPSRMKAVIYSKHFWSGQDPRSSSSKRKYGDPEAFRPNQSTGLVDMSKDRNTSRSPGNPGESYVNFDYGWFGDQKEADHNETVWTHANHYHSPDGGMGPMKVYESKFRNWSAGYADFDRGTYMITFIPKSWNTAPAKVTQGWP | Soluble | Insoluble | 0.21791129 | 0.4840098 | 0.002389605 | [0] |
| WP_012885841_59 | WP_012885841 | WP_012885841 | MEHSVIEPTVPMPMPAMFDAPSGIFDSLDDAVQAAVYAQQQLNSVELRQQVIKAIRVAGERYAQVLAEMAVAETGMGRVVDKYIKNVSQARHTPGIECLSAEVLTGDNGLTLIENAPWGVVASVTPSTNPAATVINNAISMIAAGNSVVFAPHPSAKNVSLRTISLLNKAIVATGGPENLLVSVSDPNIETAQRLFRYPGIGLLVVTGGEAVVEAARRHTDKRLIAAGAGNPPVVVDETADIPKAARAIVKGASFDNNIICADEKVLIVVDSVADALLAEMQRNHAVLLTPAQTEQLLPALLSDIDEQGKGRVNRDYVGRDATKLAEAIGLEVNEYTRLLLAETDASHPFAVTELMMPVLPVVRVKSVDDAIALALKLENGCRHTAAMHSTNIRNLNRMANAINTSIFVKNGPCIAGLGLGGEGWTSMTISTPTGEGVTSARTFVRLRRCVLVDMFRIALE | Soluble | Insoluble | 0.37573433 | 0.49152416 | 0.00213466 | [0] |
| WP_229016770.1_62 | WP_229016770.1 | WP_229016770.1 | MEQVVKAVLLKMQSSDTPPAAVHEMGVFASLDDAVAAAKIAQQGLKSVAMRQLAIAAIREAGEKHARDLAELAVSETGMGRVEDKFAKNVAQARGTPGVECLSPQVLTGDNGLTLIENAPWGVVASVTPSTNPAATVINNAISLIAAGNSVIFAPHPAAKKVSQRAITLLNQAIVAAGGPENLLVTVANPDIETAQRLFKFPGIGLLVVTGGEAVVEAARKHTNKRLIAAGAGNPPVVVDETADLARAAQSIVKGASFDNNIICADEKVLIVVDSVADELMRLMESQHAVKLTAEQAQQLQPVLLKNIDERGKGTVSRDWVGRDAAKIAAAIGLTVPEQTRLLFVETTAEHPFAVTELMMPVLPVVRVANVADAIALAVKLEGGCHHTAAMHSRNIENMNQMANAIDTSIFVKNGPCIAGLGLGGEGWTTMTITTPTGEGVTSARTFVRLRRCVLVDAFRIVLE | Soluble | Insoluble | 0.4976308 | 0.501178 | 0.002125934 | [0] |
| WP_137594799.1_63 | WP_137594799.1 | WP_137594799.1 | MNQQDIEQVVKAVLLKMLKMLKMQSSDTPPAAVHEMGVFASLDDAVAAAKIAQQGLKSVAMRQLAIAAIREAGEKHARDLAELAVSETGMGRVEDKFAKNVAQARGTPGVECLSPQVLTGDNGLTLIENAPWGVVASVTPSTNPAATVINNAISLIAAGNSVIFAPHPAAKKVSQRAITLLNQAIVAAGGPENLLVTVANPDIETAQRLFKFPGIGLLVVTGGEAVVEAARKHTNKRLIAAGAGNPPVVVDETADLARAAQSIVKGASFDNNIICADEKVLIVVDSVADELMRLMEGQHAVKLTAEQAQQLQPVLLKNIDERGKGTVSRDWVGRDAGKIAAAIGLKVPQEMRLLFVETTAEHPFAVTELMMPVLPVVRVANVADAIALAVKLEGGCHHTAAMHSRNIENMNQMANAIDTSIFVKNGPCIAGLGLGGEGWTTMTITTPTGEGVTSARTFVRLRRCVLVDAFRIVLE | Soluble | Insoluble | 0.45814896 | 0.50118655 | 0.00210083 | [0] |
| NHX18690.1_64 | NHX18690.1 | NHX18690.1 | MVKAVLLKMQSSDTPSAAVHEMGVFASLDDAVAAAKVAQQGLKSVAMRQLAIAAIREAGEKHARDLAELAVSETGMGRVEDKFAKNVAQARGTPGVECLSPQVLTGDNGLTLIENAPWGVVASVTPSTNPAATVINNAISLIAAGNSVIFAPHPAAKKVSQRAITLLNQAIVAAGGPENLLVTVANPDIETAQRLFKFPGIGLLVVTGGEAVVEAARKHTNKRLIAAGAGNPPVVVDETADLARAAQSIVKGASFDNNIICADEKVLIVVDSVADELMRLMEGQHAVKLTAEQAQQLQPVLLKNIDERGKGTVSRDWVGRDAGKIAAAIGLKVPQETRLLFVETTAEHPFAVTELMMPVLPVVRVANVADAIALAVKLEGGCHHTAAMHSRNIENMNQMANAIDTSIFVKNGPCIAGLGLGGEGWTTMTITTPTGEGVTSARTFVRLRRCVLVDAFRIVLE | Soluble | Insoluble | 0.4729565 | 0.50118345 | 0.002141817 | [0] |
| WP_232921703.1_65 | WP_232921703.1 | WP_232921703.1 | MDRQQIEQVVKAVLAGMAANSAPEPVTPPCGTGVFASLDDAVQAASVAQKALTSVAMRQKVVAAIRLAGEQHAQQLAEMAVAETGMGRVADKCAKNIAQARGTPGVECLTPQVLTGDNGLTLIENAPWGVVASVTPSTNPAATVINNAISMISAGNSVVFAPHPAAKKVSQQTITWLNEAIVAAGGPANLLVTVLNPDIETAQRLFKYPGIGLLVVTGGEAVVDAARKHTNKRLIAAGAGNPPVVVDETADIPRAAKAIVQGASFDNNIICADEKVLIVVESVADALLEEMQRNHAVLLSDAQAEKLLPVLLKNVDEQGKGQVCRDWVGRDATKIAAAIDLNVASETRLLLVQTTARHPFAVTEMMMPVLPMIRVANVSEAIALAVKLEGGCHHTAAMHSRNIDNMNAMANAIDTSIFVKNGPCIAGLGLGGEGWTSMTITTPTGEGVTSARTFVRLRRCVLVDGFRIVLE | Soluble | Insoluble | 0.4443432 | 0.5011925 | 0.002118686 | [0] |
| WP_225622697.1_66 | WP_225622697.1 | WP_225622697.1 | MVPMPTPVIFDAPSGIFDSLDDAVQAAAQAQQQLTSVELRQQVIKAIRVAGERYAQVLAEMAVAETGMGRVVDKYIKNVSQARHTPGIECLSAEVLTGDNGLTLIENAPWGVVASVTPSTNPAATVINNAISMIAAGNSVVFAPHPSAKKVSLRTISLLNKAIVATGGPENLLVSVADPNIETAQRLFRYPGIGLLVVTGGEAVVEAARKHTDKRLIAAGAGNPPVVVDETADIPKAARAIVKGASFDNNIICADEKVLIVVDSVADALLAEMQRNHAVLLTPEQTERLLPVLLSDIDAQGKGRVNRDYVGRDATKLAAAIGLEVNEYTRLLLAETDASHPFAVTELMMPVLPVVRVKNVDDAIALAITLENGCRHTAAMHSTNIRNLNRMANAINTSIFVKNGPCIAGLGLGGEGWTSMTISTPTGEGVTSARTFVRLRRCVLVDMFRIALE | Soluble | Insoluble | 0.38709843 | 0.5011814 | 0.002162271 | [0] |
| WP_320763852.1_67 | WP_320763852.1 | WP_320763852.1 | MEQSTLELNAPARPVADFDAQYGIFASLDDAVSAATLAQKRLDNVIVRQNVITAIRQAGERYAQVLAEMAVEETGMGRVADKYAKNVSQARSTPGTESLSAKVVTGDNGLTLIENAPWGVVASVTPSTNPAATVINNAISMIAAGNSIVFAPHPSAKKVSLYTISLLNKAIVTAGGPENLLVTVADPNIETAQRLFRHPGISLLVVTGGEAVVEAARKHTDKRLIAAGAGNPPVVVDETANIERAARDIVRGASFDNNIICVDEKVAIVVNSVADELLSEMQYHQAVLLTRQQIEQLQSLLLTDIDEQGRGRPHRDWVGRDAAKIAAAVGLNVSEHTRLLLAETDANHPFAVTEMMMPVLPIVRVRDVEEAIELAIHLEGGRRHTAAMHSTNINNLHKMANAINTSIFVKNGPCIAGLGLGGEGCTSMTISTPTGEGVTSARTFVRIRRCVMVDMFRIVLE | Soluble | Insoluble | 0.3902372 | 0.49824578 | 0.002142548 | [0] |
| WP_049779824.1_68 | WP_049779824.1 | WP_049779824.1 | MPMPAMFDAPSGIFDSLDDAVQAAVYAQQQLNSVELRQQVIKAIRVAGERYAQVLAEMAVAETGMGRVVDKYIKNVSQARHTPGIECLSAEVLTGDNGLTLIENAPWGVVASVTPSTNPAATVINNAISMIAAGNSVVFAPHPSAKNVSLRTISLLNKAIVATGGPENLLVSVSDPNIETAQRLFRYPGIGLLVVTGGEAVVEAARRHTDKRLIAAGAGNPPVVVDETADIPKAARAIVKGASFDNNIICADEKVLIVVDSVADALLAEMQRNHAVLLTPAQTEQLLPALLSDIDEQGKGRVNRDYVGRDATKLAEAIGLEVNEYTRLLLAETDASHPFAVTELMMPVLPVVRVKSVDDAIALALKLENGCRHTAAMHSTNIRNLNRMANAINTSIFVKNGPCIAGLGLGGEGWTSMTISTPTGEGVTSARTFVRLRRCVLVDMFRIALE | Soluble | Insoluble | 0.3880624 | 0.50118846 | 0.002169684 | [0] |
| WP_240947156.1_69 | WP_240947156.1 | WP_240947156.1 | MPMPAMFDAPSGIFDSLDDAVQAATYAQQQLNSVELRQQVIKAIRVAGERYAQVLAEMAVAETGMGRVVDKYIKNVSQARHTPGIECLSAEVLTGDNGLTLIENAPWGVVASVTPSTNPAATVINNAISMIAAGNSVVFAPHPSAKNVSLRTISLLNKAIVATGGPENLLVSVSDPNIETAQRLFRYPGIGLLVVTGGEAVVEAARKHTDKRLIAAGAGNPPVVVDETADIPKAARAIVKGASFDNNIICADEKVLIVVDSVADALLAEMQRNHAVLLTPAQTEQLLPALLSDIDEQGKGRVNRDYVGRDASKLAEAIGLEVNEYTRLLLAETDASHPFAVTELMMPVLPVVRVKNVDEAIALALKLENGCRHTAAMHSTNIRNLNRMANAINTSIFVKNGPCIAGLGLGGEGWTSMTISTPTGEGVTSARTFVRLRRCVLVDMFRIALE | Soluble | Insoluble | 0.39403674 | 0.5011895 | 0.00216959 | [0] |
